# Supplementary figures and images for: Single-nucleus atlas of the Artemia female reproductive system suggests germline repression of the Z chromosome
Source: PLoS Genet. 2024 Aug 30;20(8):e1011376. doi: 10.1371/journal.pgen.1011376 (PMC11392275; doi:10.1371/journal.pgen.1011376)

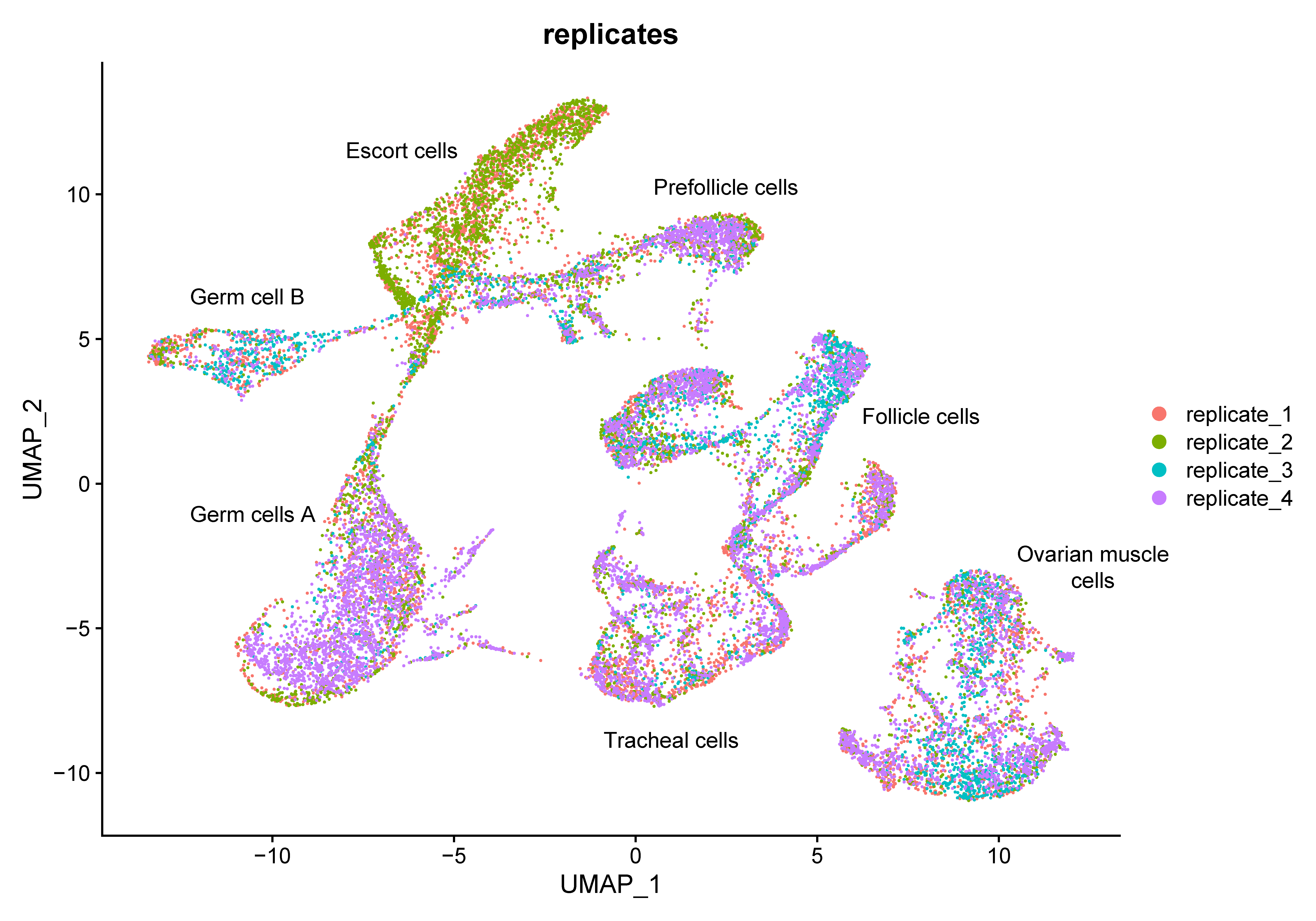

Supplement: S1 Fig — (TIF) [file pgen.1011376.s001.tif]

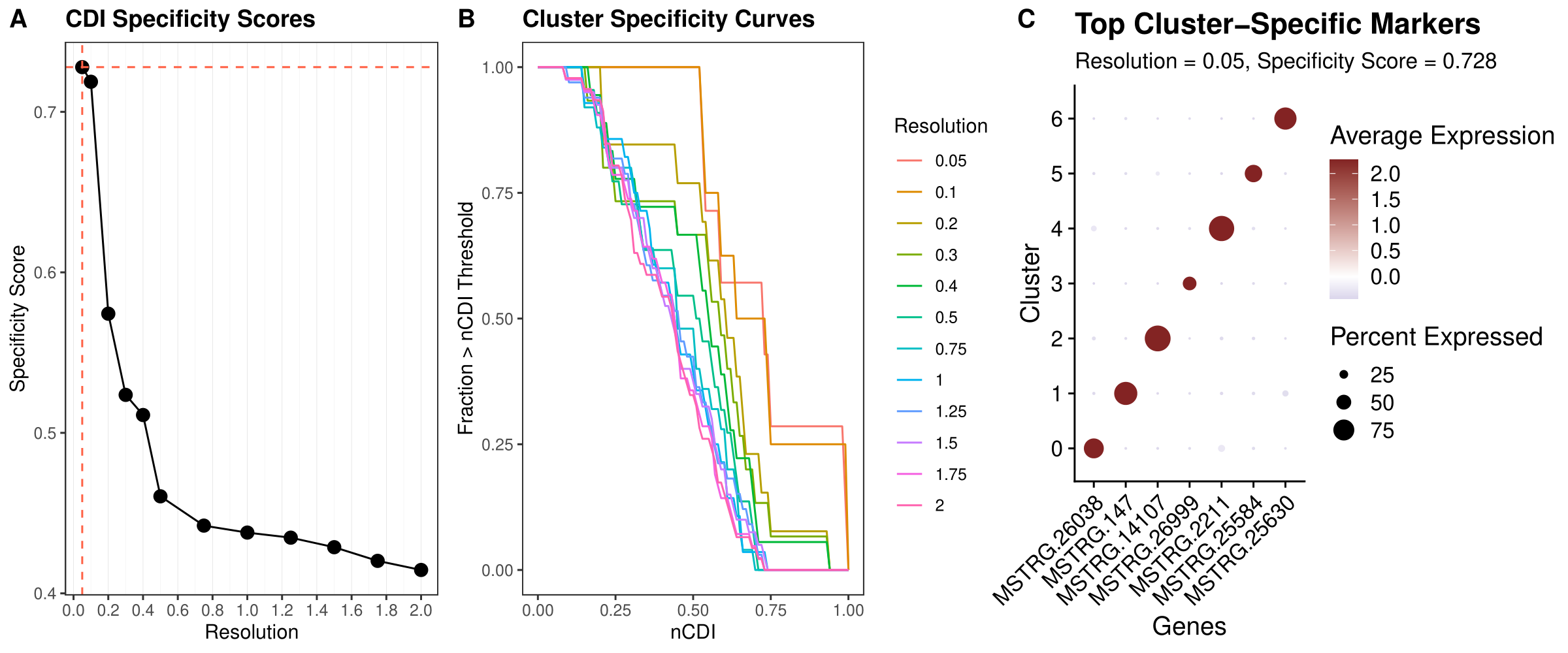

Supplement: S2 Fig — A) specificity scores. B) Specificity curves. C) Dot plot of top cluster-specific markers. (TIFF) [file pgen.1011376.s002.tiff]

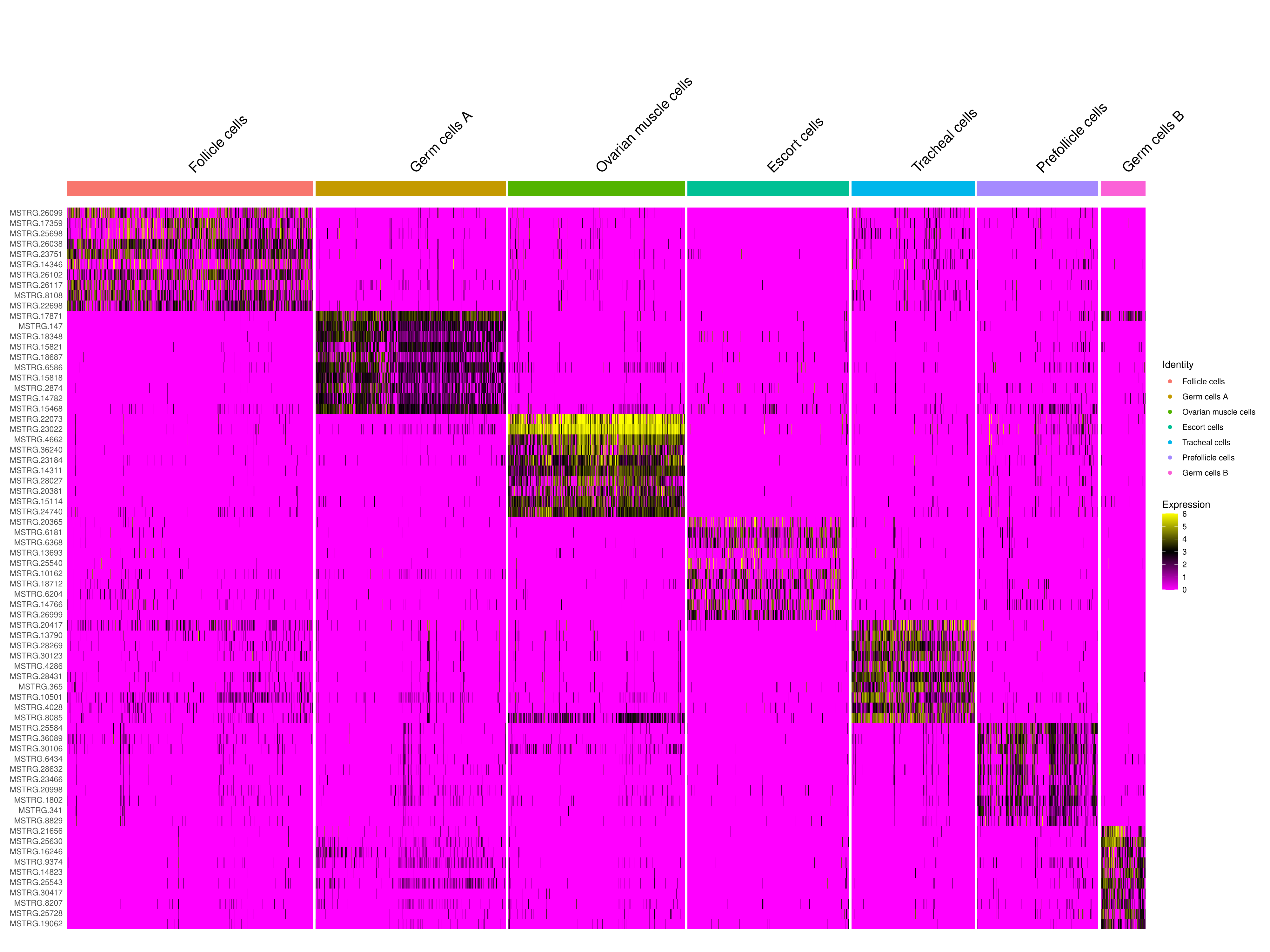

Supplement: S3 Fig — (TIFF) [file pgen.1011376.s003.tiff]

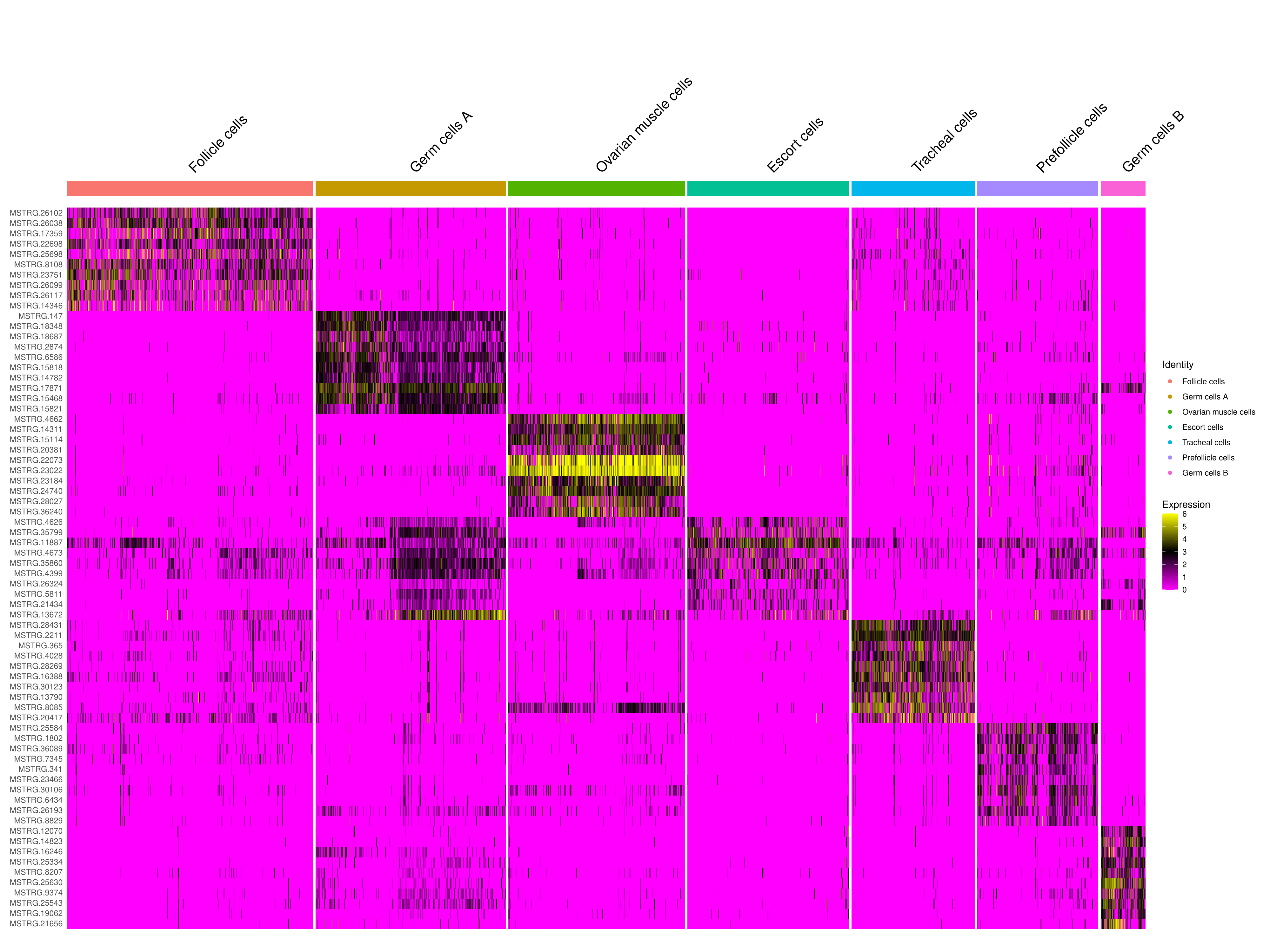

Supplement: S4 Fig — (TIFF) [file pgen.1011376.s004.tiff]

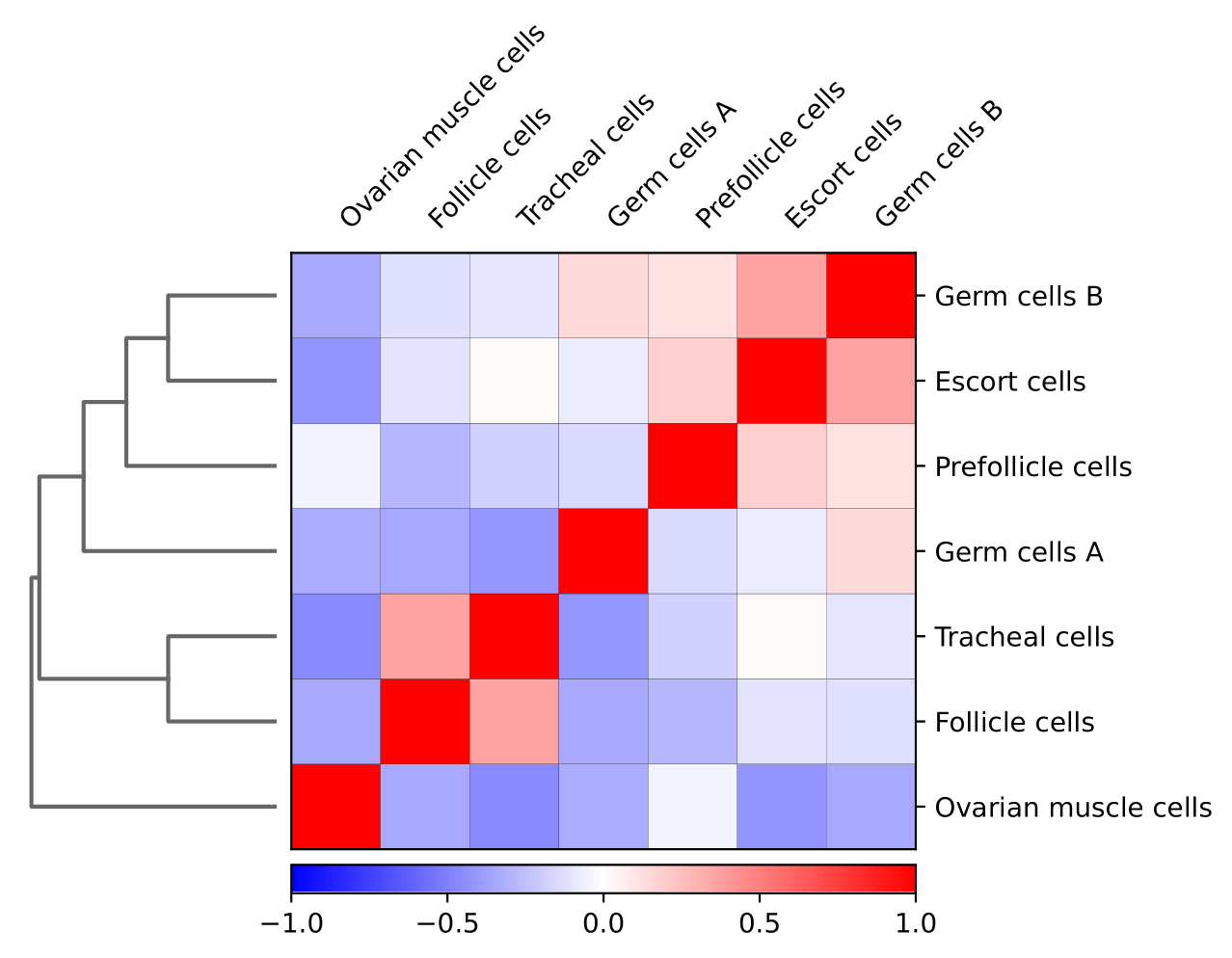

Supplement: S5 Fig — (TIFF) [file pgen.1011376.s005.tiff]

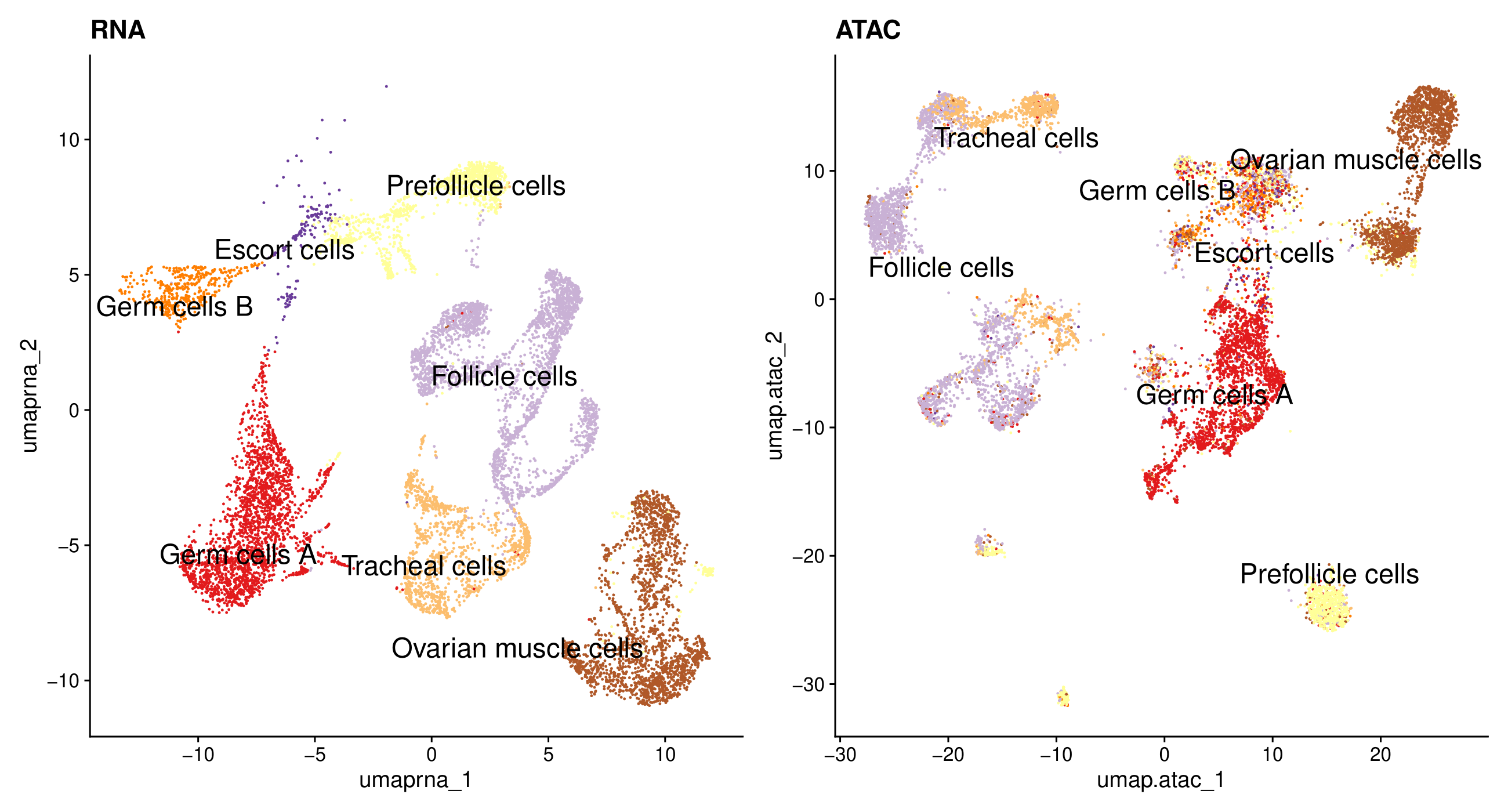

Supplement: S6 Fig — A) UMAP of replicates 3 and 4 nuclei based on expression. B) UMAP of the nuclei from replicates 3 and 4 nuclei based on peaks. (TIFF) [file pgen.1011376.s006.tiff]

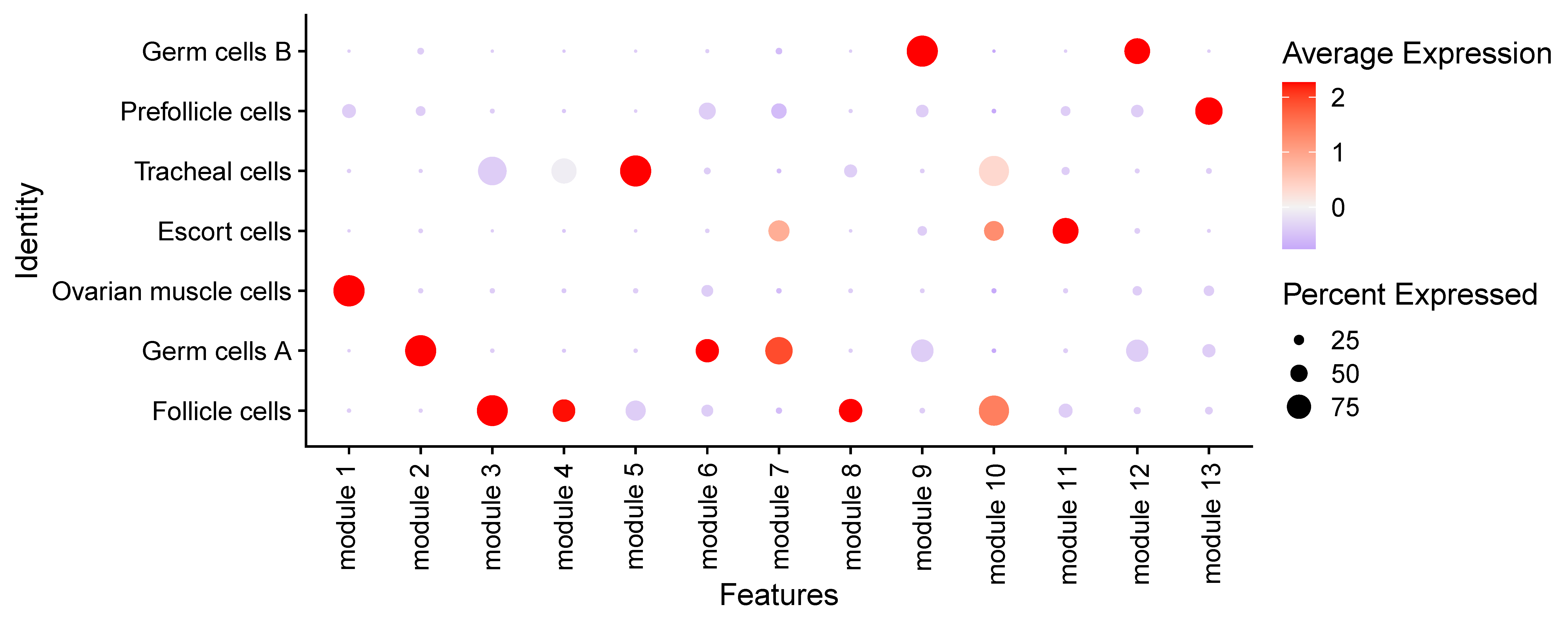

Supplement: S7 Fig — (TIF) [file pgen.1011376.s007.tif]

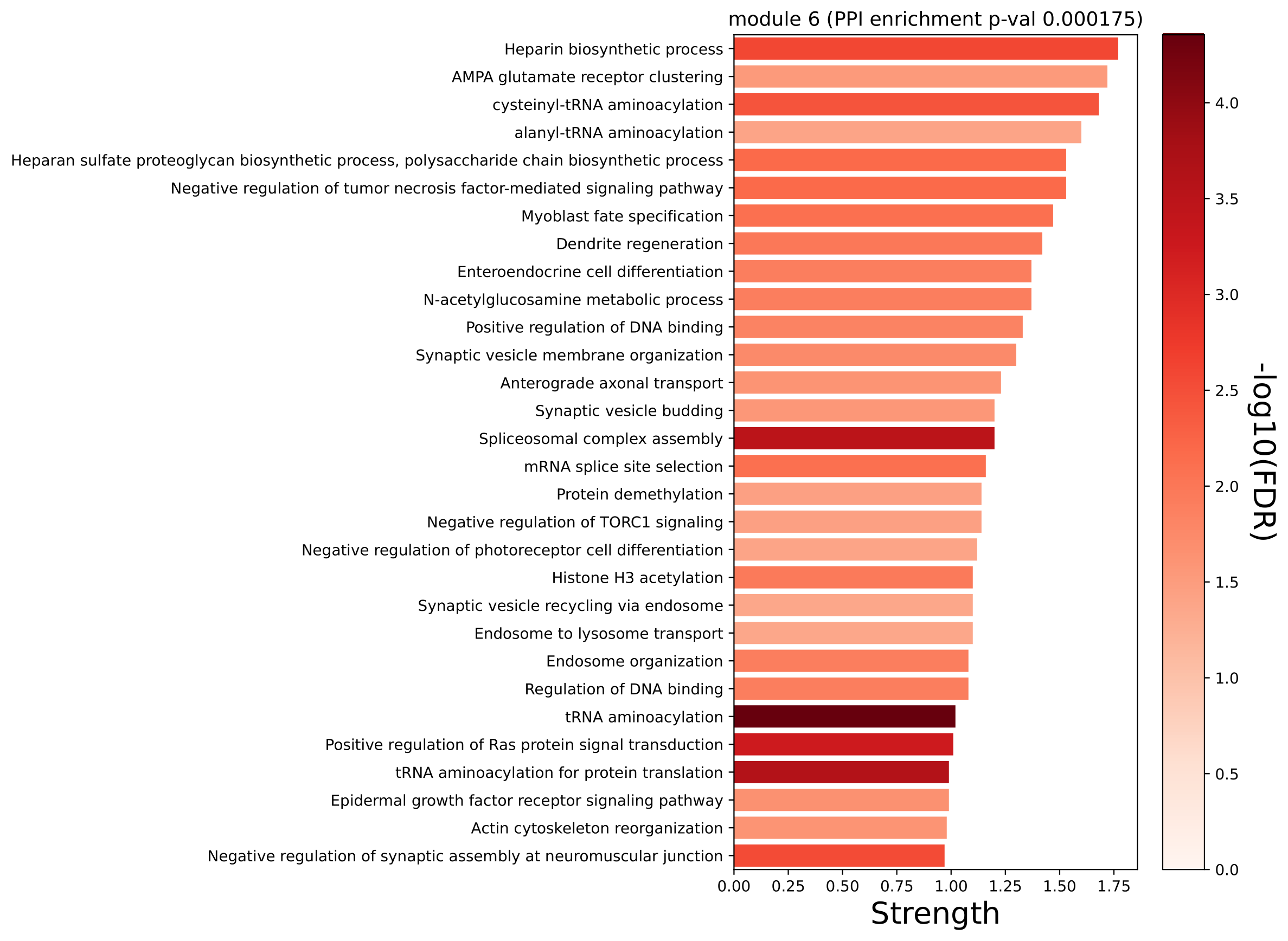

Supplement: S8 Fig — (TIFF) [file pgen.1011376.s008.tiff]

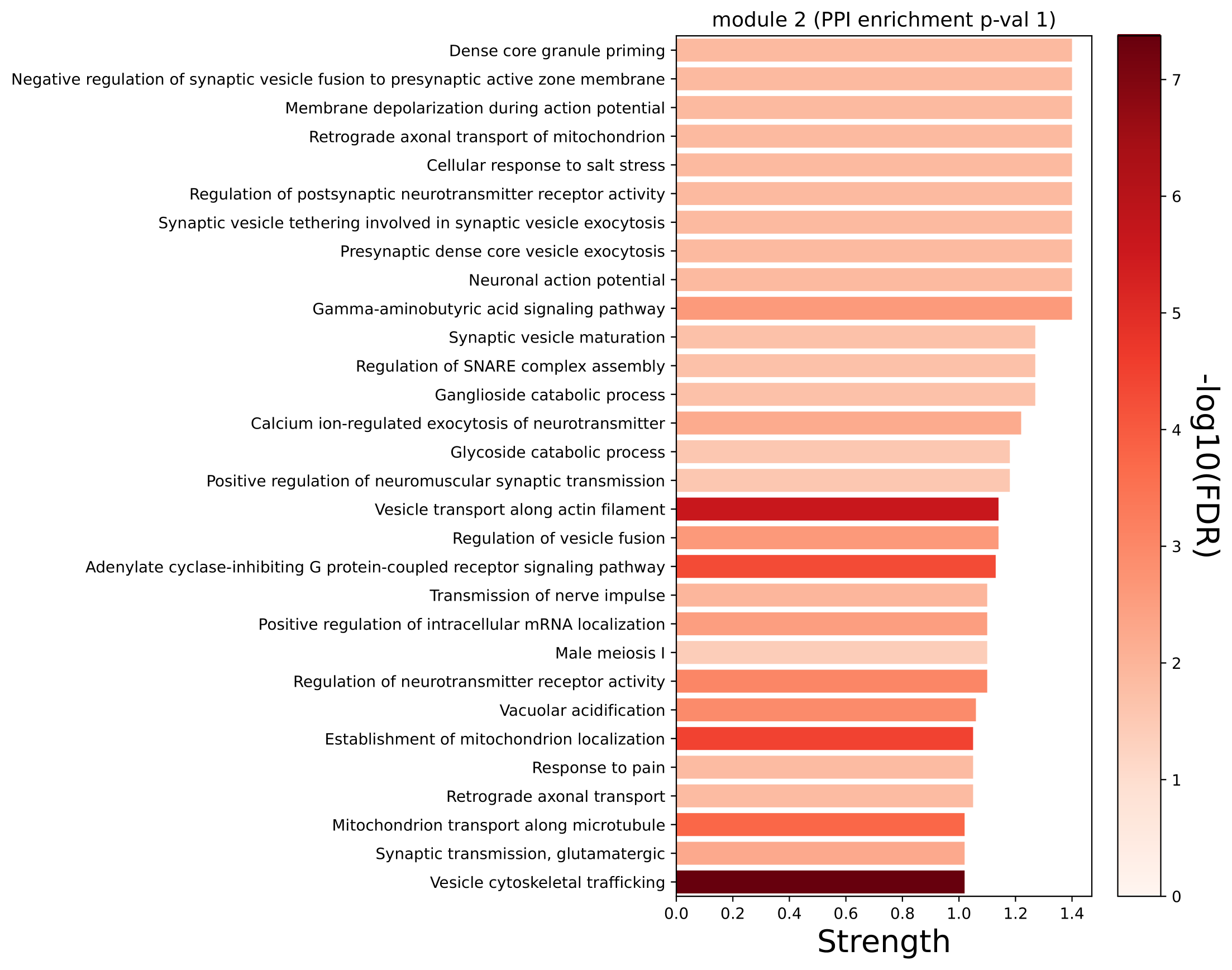

Supplement: S9 Fig — (TIFF) [file pgen.1011376.s009.tiff]

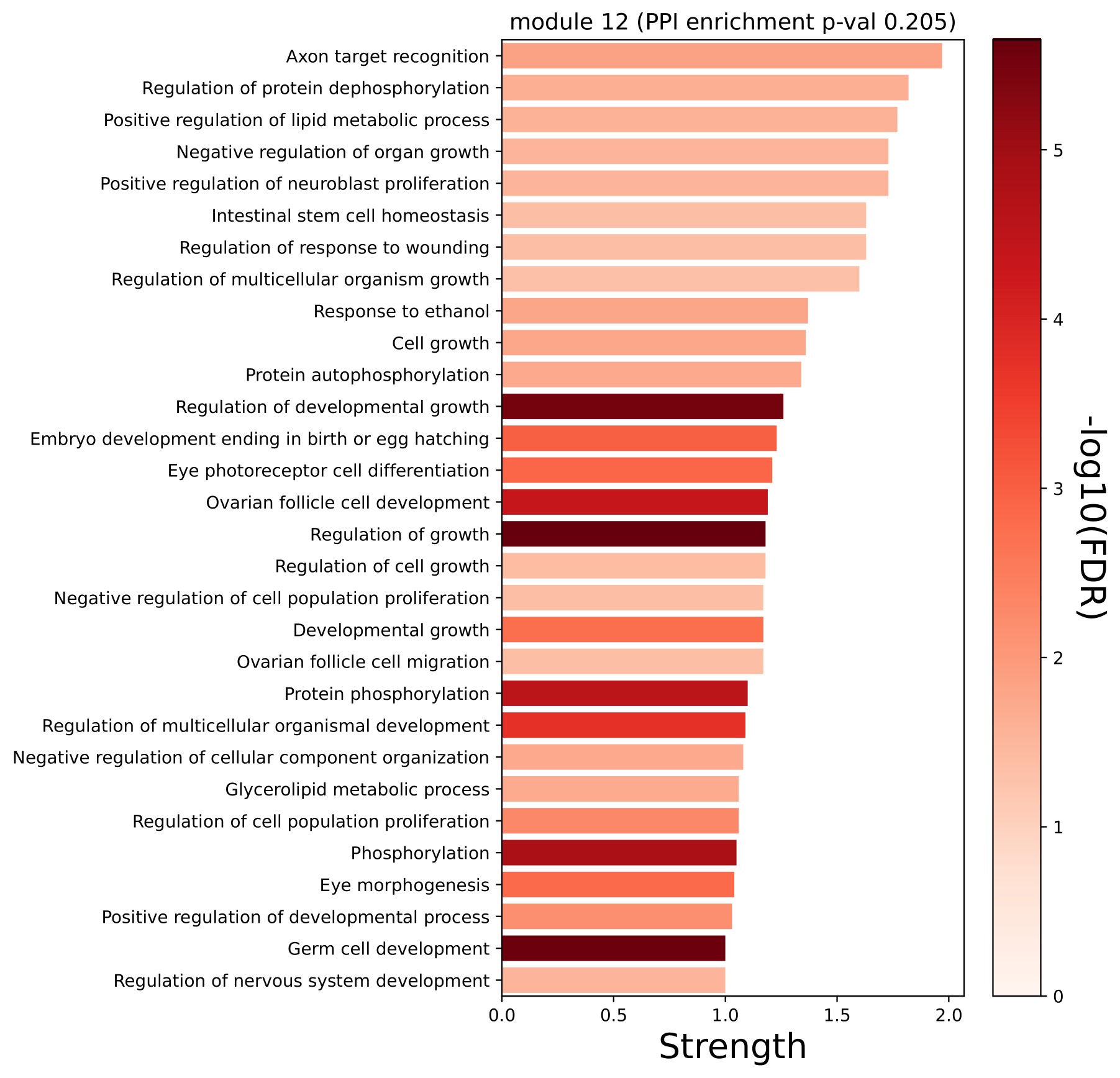

Supplement: S10 Fig — (TIFF) [file pgen.1011376.s010.tiff]

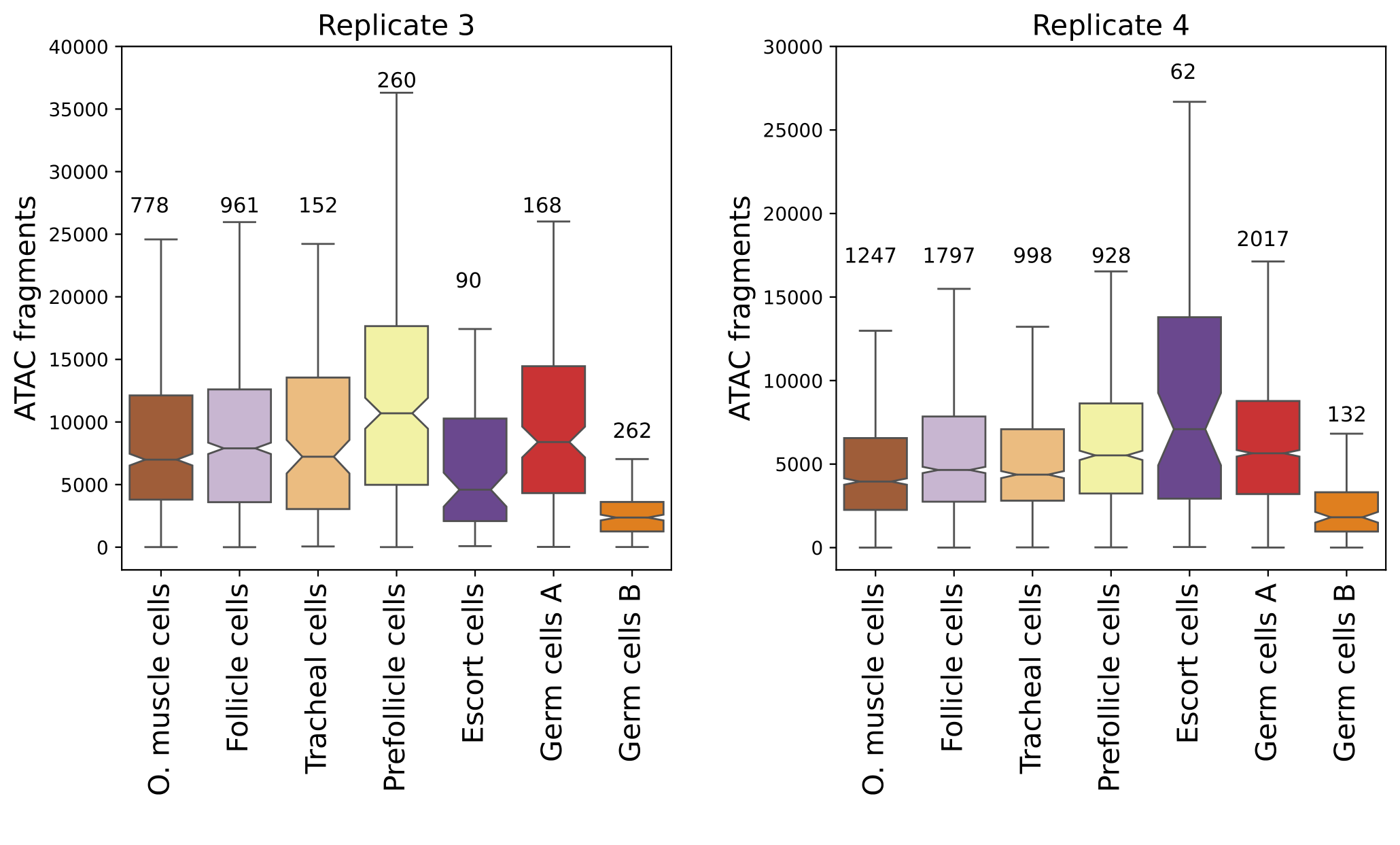

Supplement: S11 Fig — A) ATAC fragments per cell for all clusters in Replicate 3. B) ATAC fragments per cell for all clusters in Replicate 4. (TIFF) [file pgen.1011376.s011.tiff]

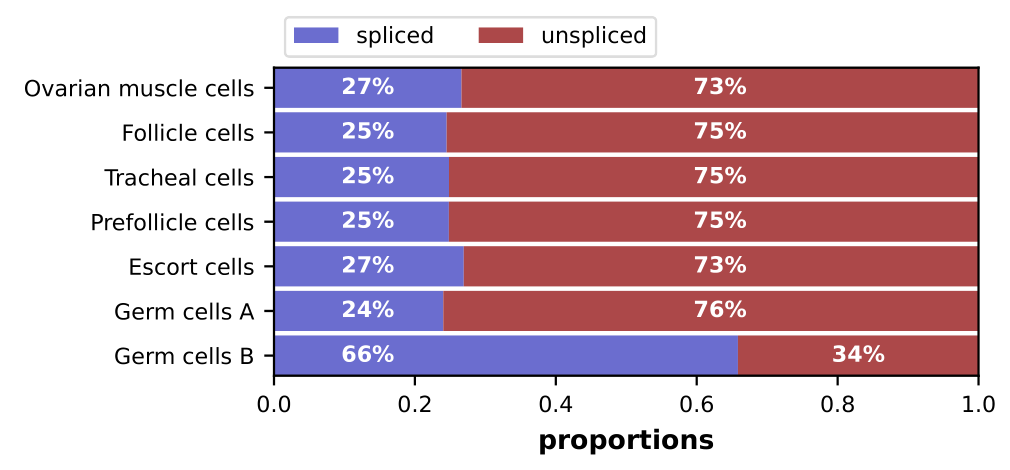

Supplement: S12 Fig — (TIFF) [file pgen.1011376.s012.tiff]

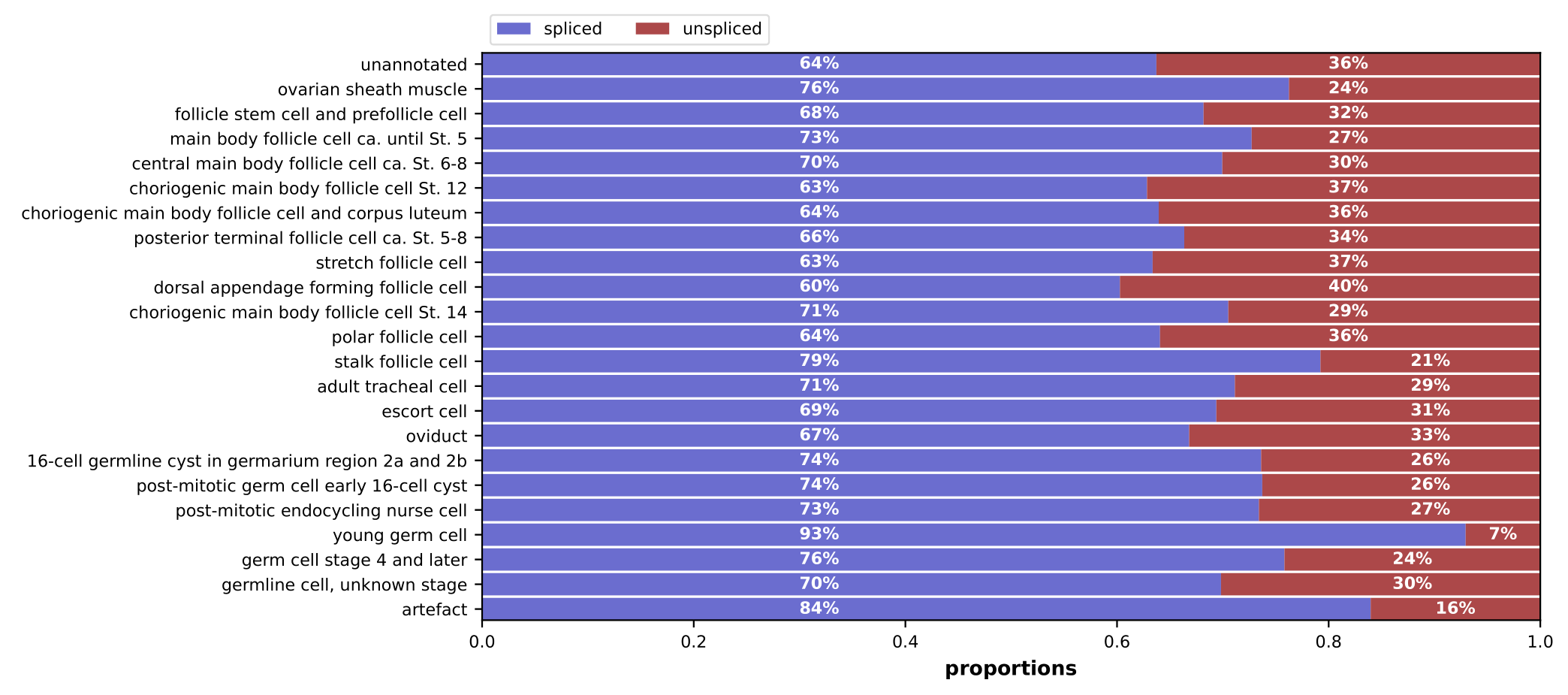

Supplement: S13 Fig — (TIFF) [file pgen.1011376.s013.tiff]

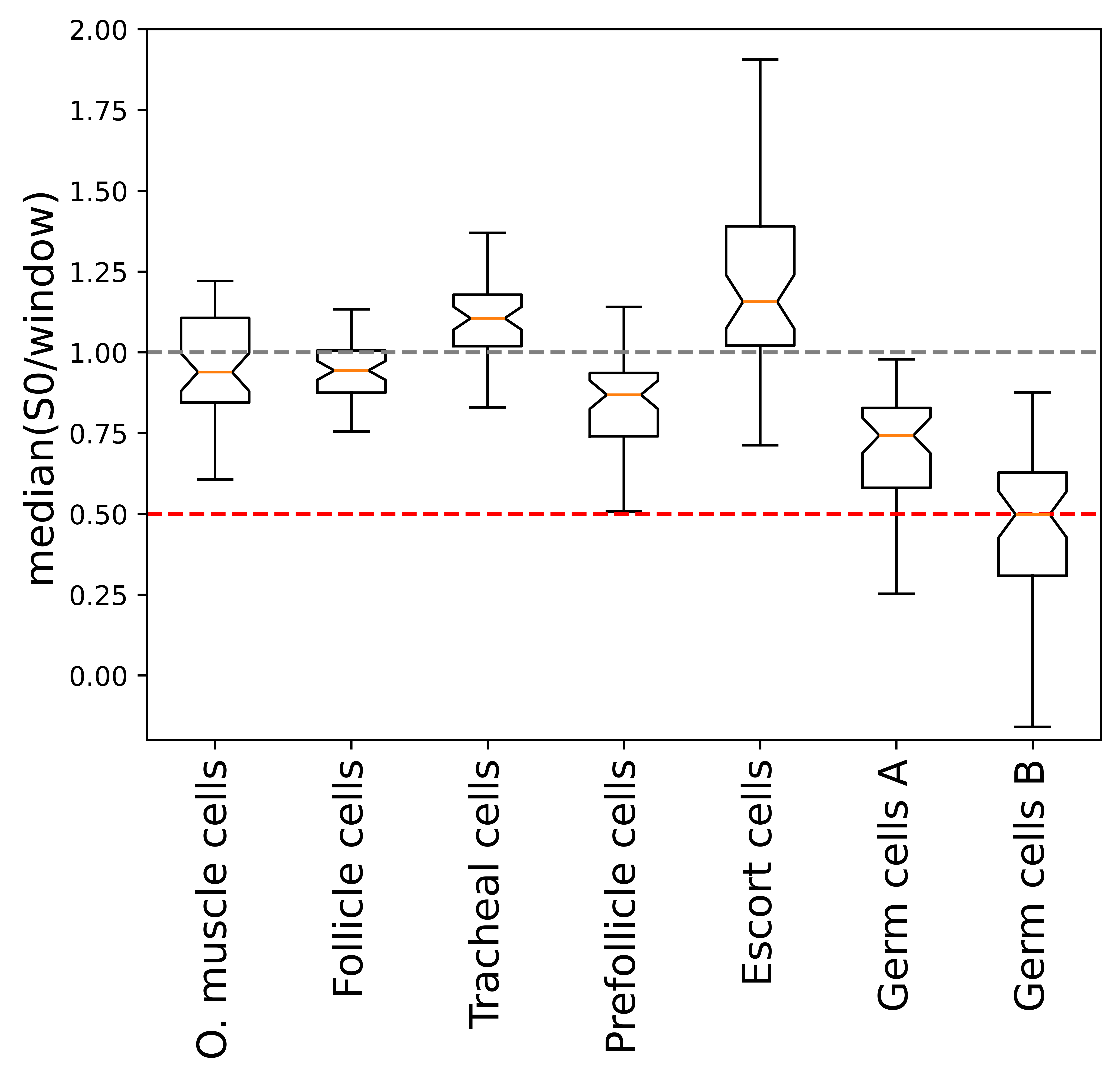

Supplement: S14 Fig — (TIFF) [file pgen.1011376.s014.tiff]

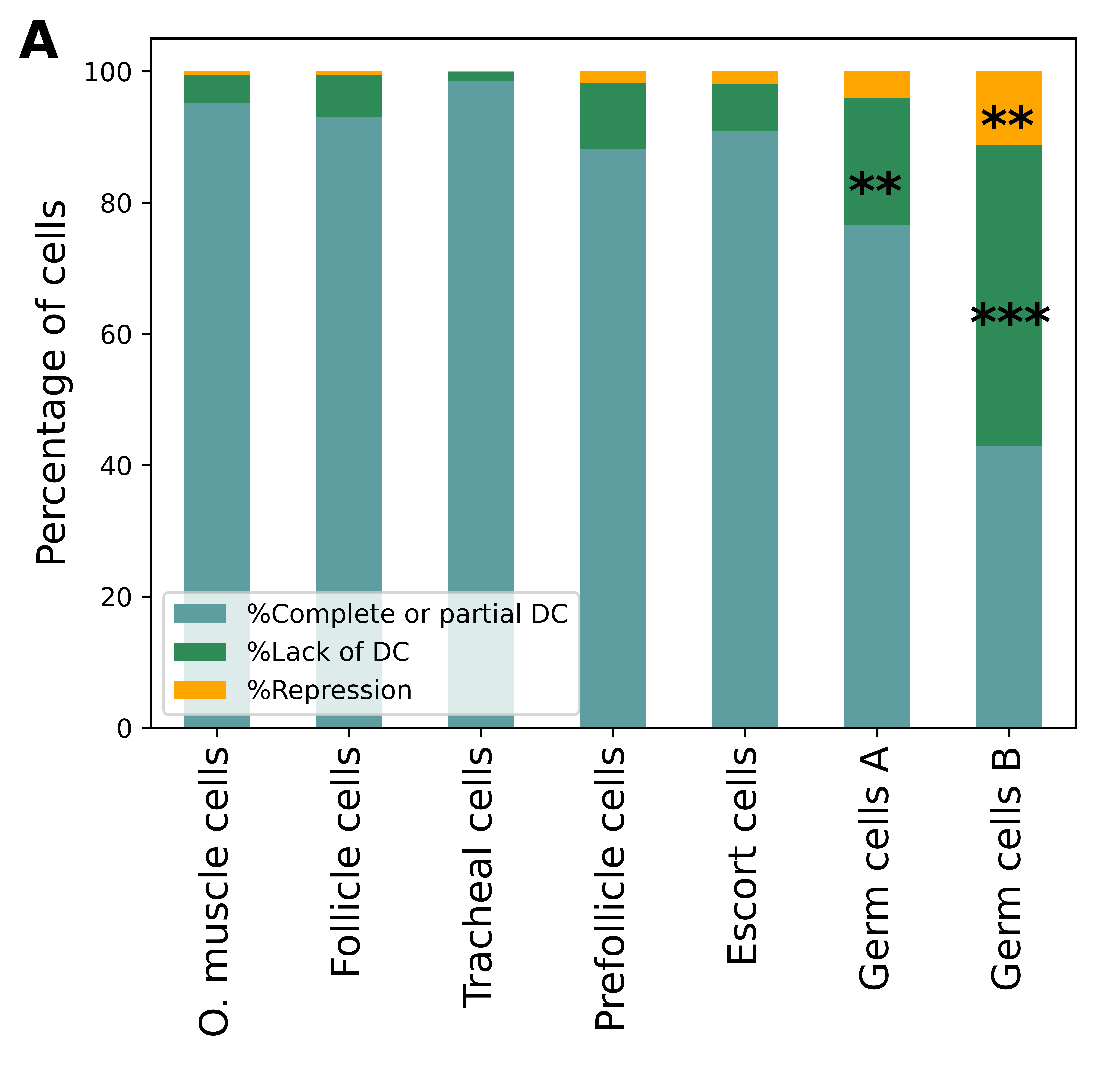

Supplement: S15 Fig — The stars show the significance values comparing group 1 clusters and somatic clusters (%Lack dosage compensation vs rest, and %Repression vs rest using Chi-square contingency test). For the stars, *** denotes p-value < = 0.001, ** denotes p-value < = 0.01, and * denotes p-value < = 0.05. (TIFF) [file pgen.1011376.s015.tiff]

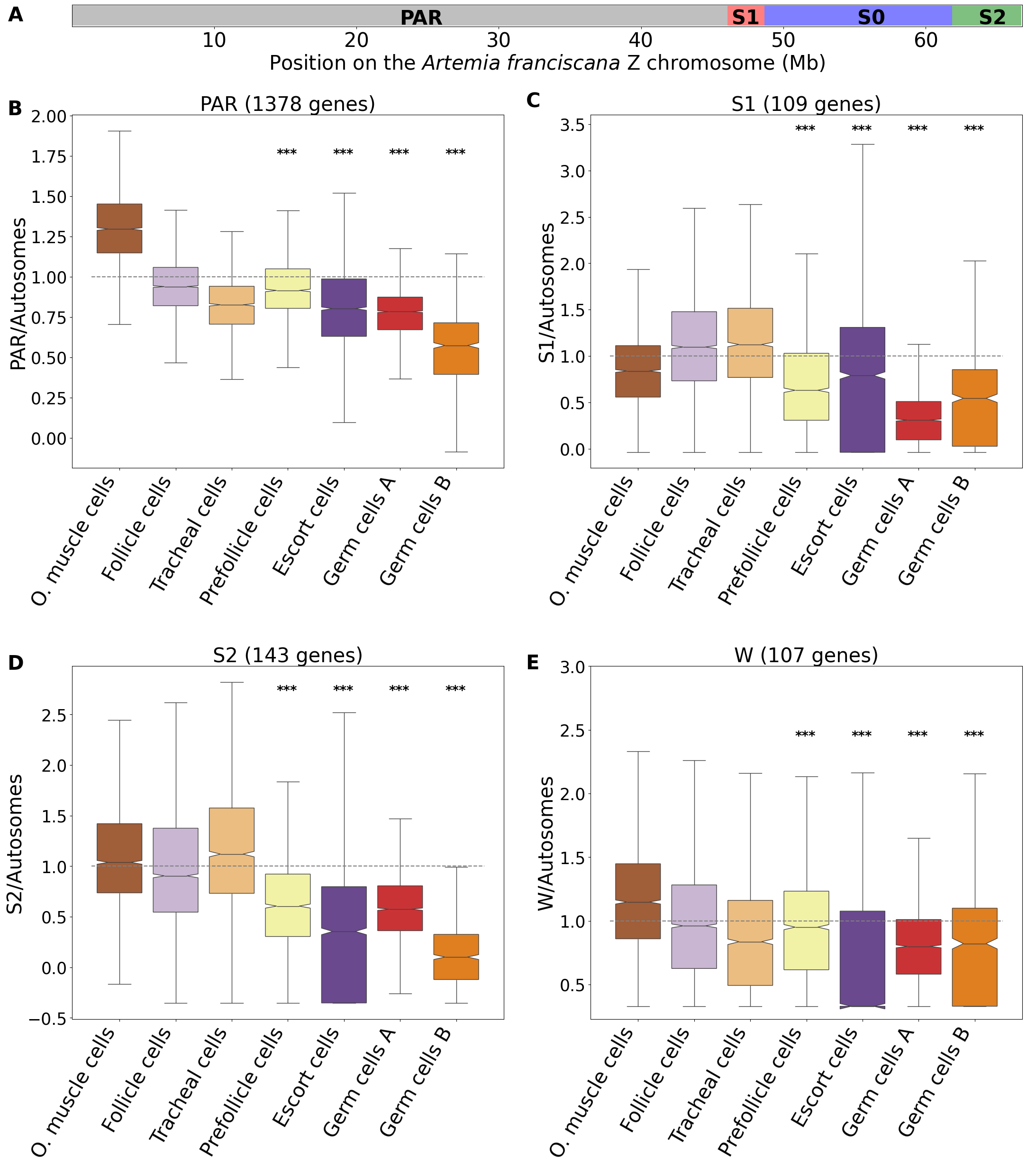

Supplement: S16 Fig — A) The structure of the Z chromosome as described in (Bett et al., 2024), with the large pseudoautosomal region (PAR), the differentiated region (S0), and two younger strata (S1 and S2). B) PAR/Autosomes expression per cell C) S1/Autosomes expression per cell D) S2/Autosomes expression per cell E) W/Autosomes expression per cell. The normalized counts matrix was used for all the estimates. (TIFF) [file pgen.1011376.s016.tiff]

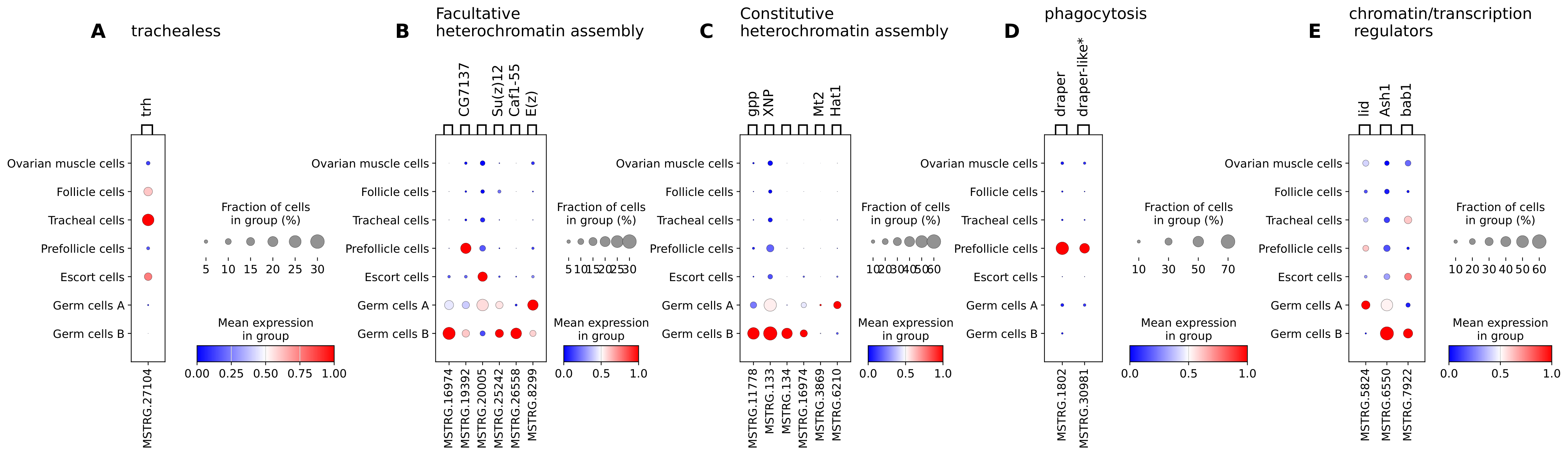

Supplement: S17 Fig — A) Trachealess expression dot plot. B) Facultative heterochromatin assembly network expression. C) Constitutive heterochromatin assembly network expression D) phagocytosis genes expression. E) Genes involved in the modeling of oocyte chromatin. (TIFF) [file pgen.1011376.s017.tiff]

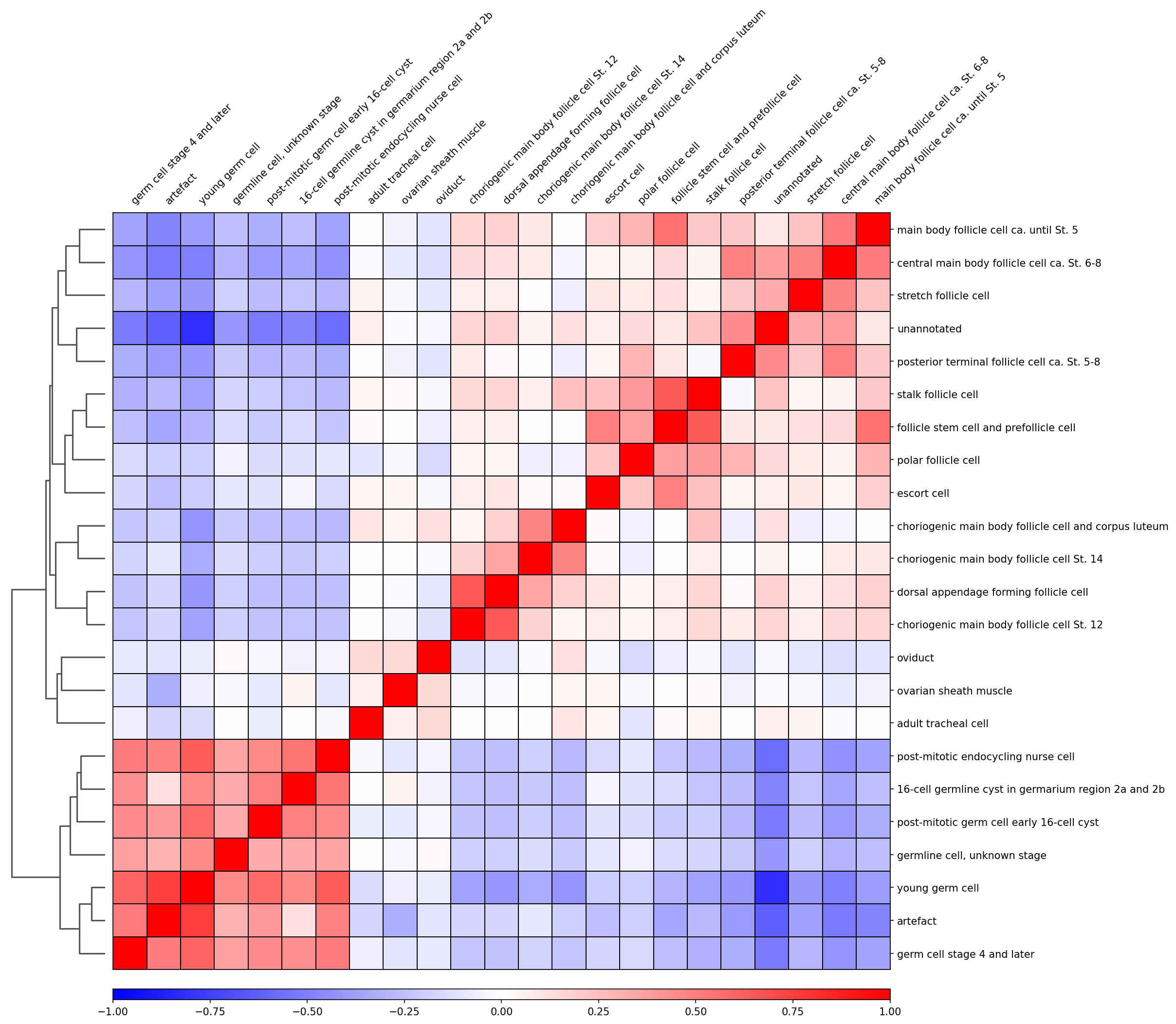

Supplement: S18 Fig — (TIFF) [file pgen.1011376.s018.tiff]

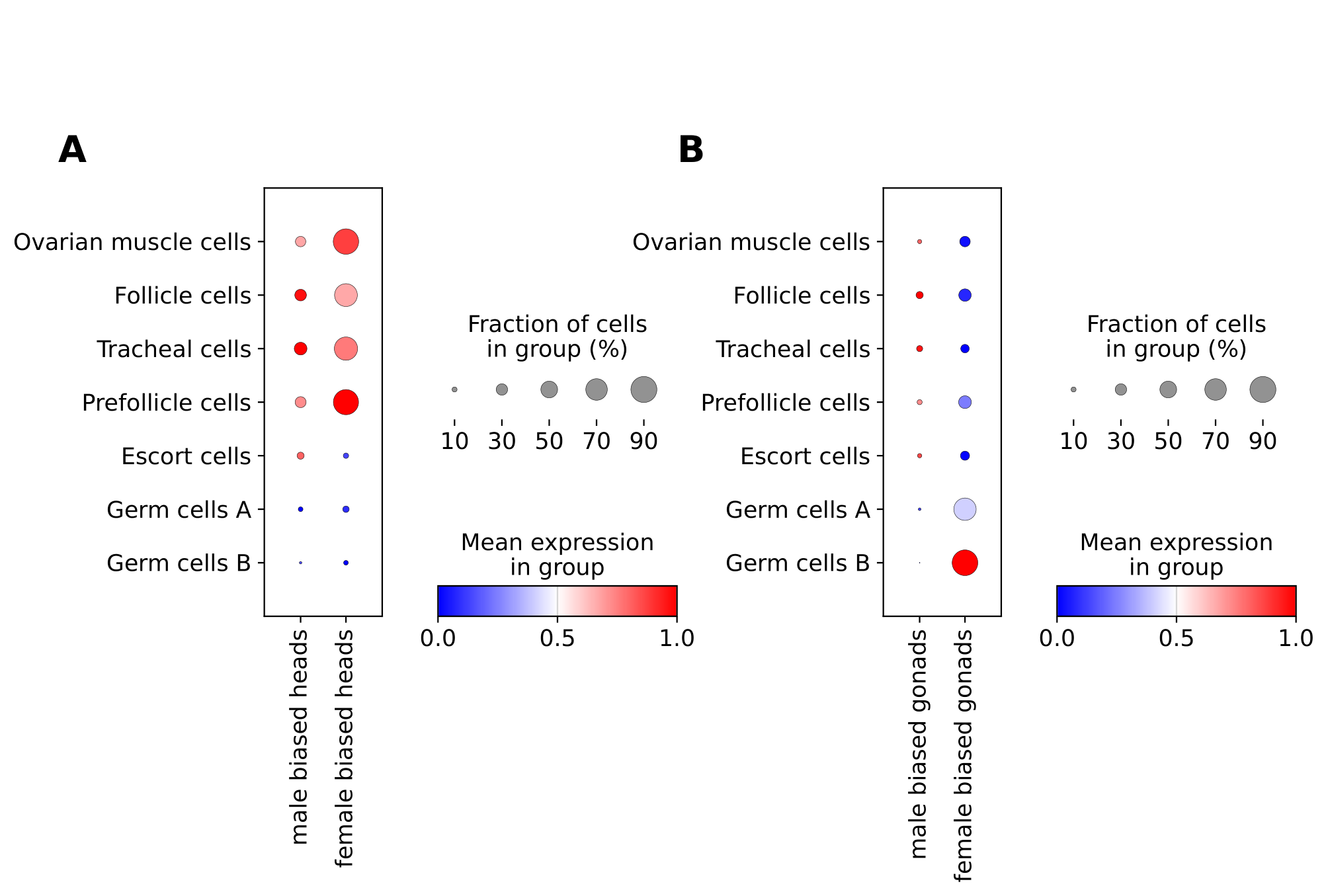

Supplement: S19 Fig — Male and female-biased genes were inferred by running DEseq2 with standard parameters on the bulk RNA-seq data from (Huylmans et al., 2019). A) Differentially expressed male vs female heads (Filtered for >5,-5 fold change and <0.01 qval). B) Differentially expressed ovaries vs testes (Filtered for >10,-10 fold change and <0.01 qval). (TIFF) [file pgen.1011376.s019.tiff]

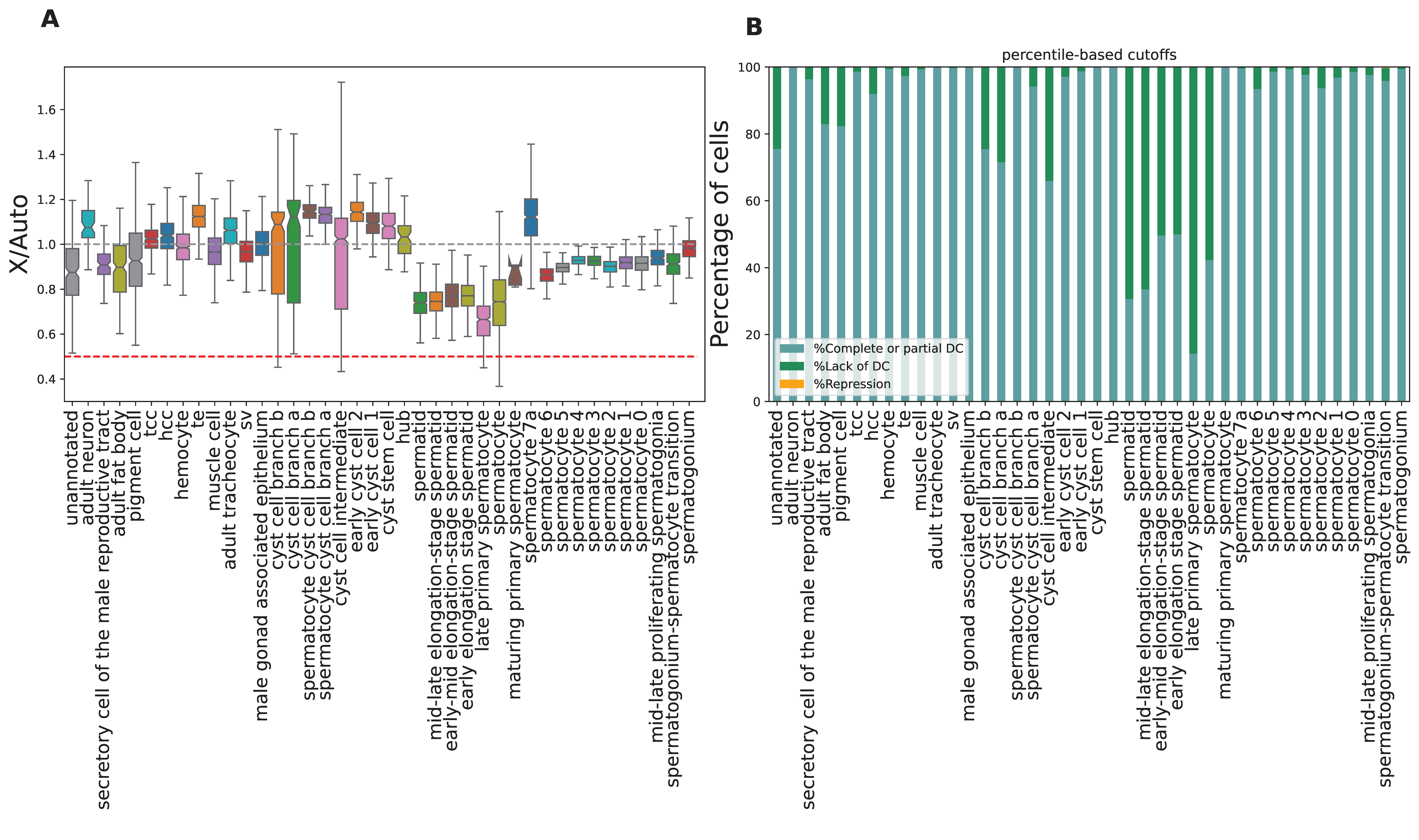

Supplement: S20 Fig — A) The X/Autosomes expression per cell estimated using the normalized counts matrix. B) The percentage of cells that have partial or complete dosage compensation (Complete or partial DC), lack dosage compensation (Lack of DC), or repression using the percentile-based cutoffs. The testis snRNA-seq data (Raz AA et al., 2023) was obtained from: https://datadryad.org/stash/dataset/doi:10.5061/dryad.m63xsj454. (TIF) [file pgen.1011376.s020.tif]

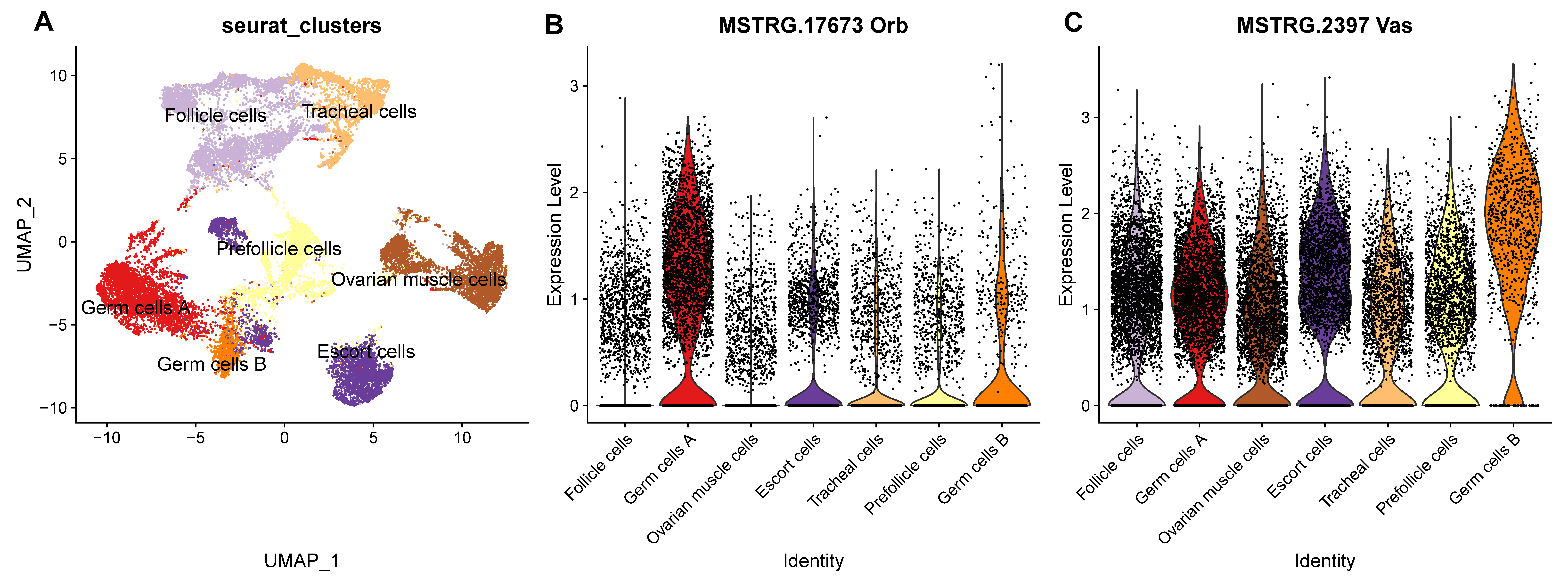

Supplement: S21 Fig — A) UMAP with no ambient RNA removal. B) Orb expression no ambient RNA removal. C) Vasa expression with no ambient RNA removal. The cells were labeled based on the annotation from the main analysis. (TIFF) [file pgen.1011376.s021.tiff]

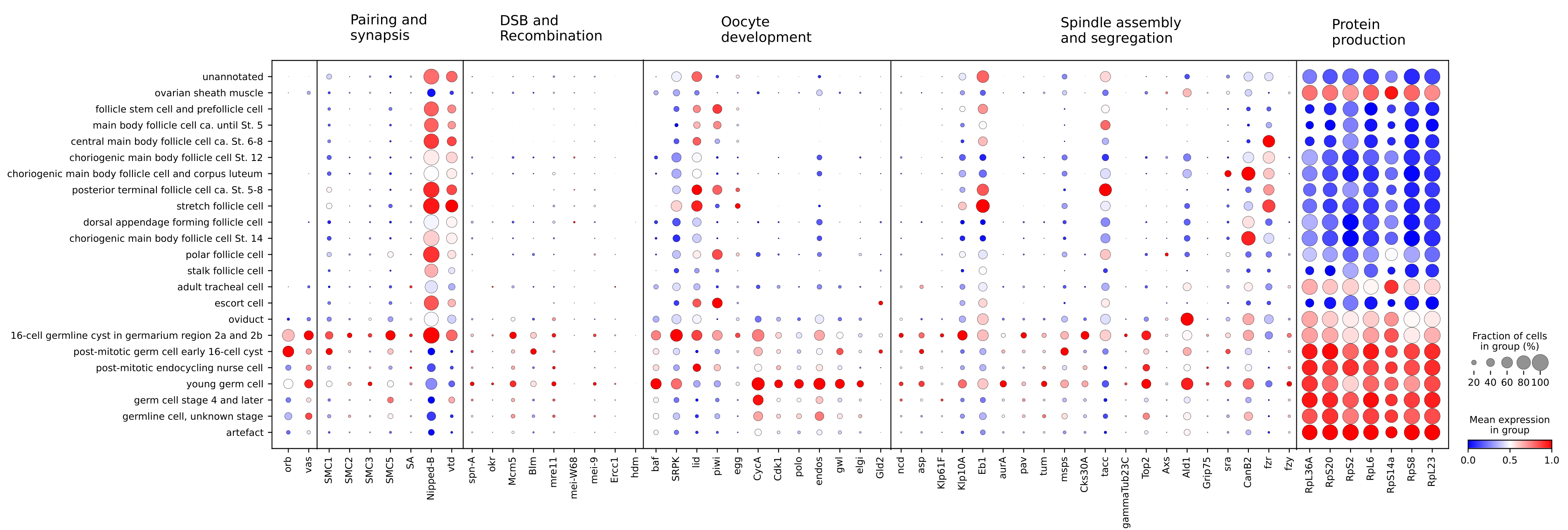

Supplement: S24 Fig — The plot was produced with Scanpy using the Fly Cell Atlas ovary dataset (10x, Stringent, H5AD, downloaded from https://flycellatlas.org/). (TIFF) [file pgen.1011376.s024.tiff]

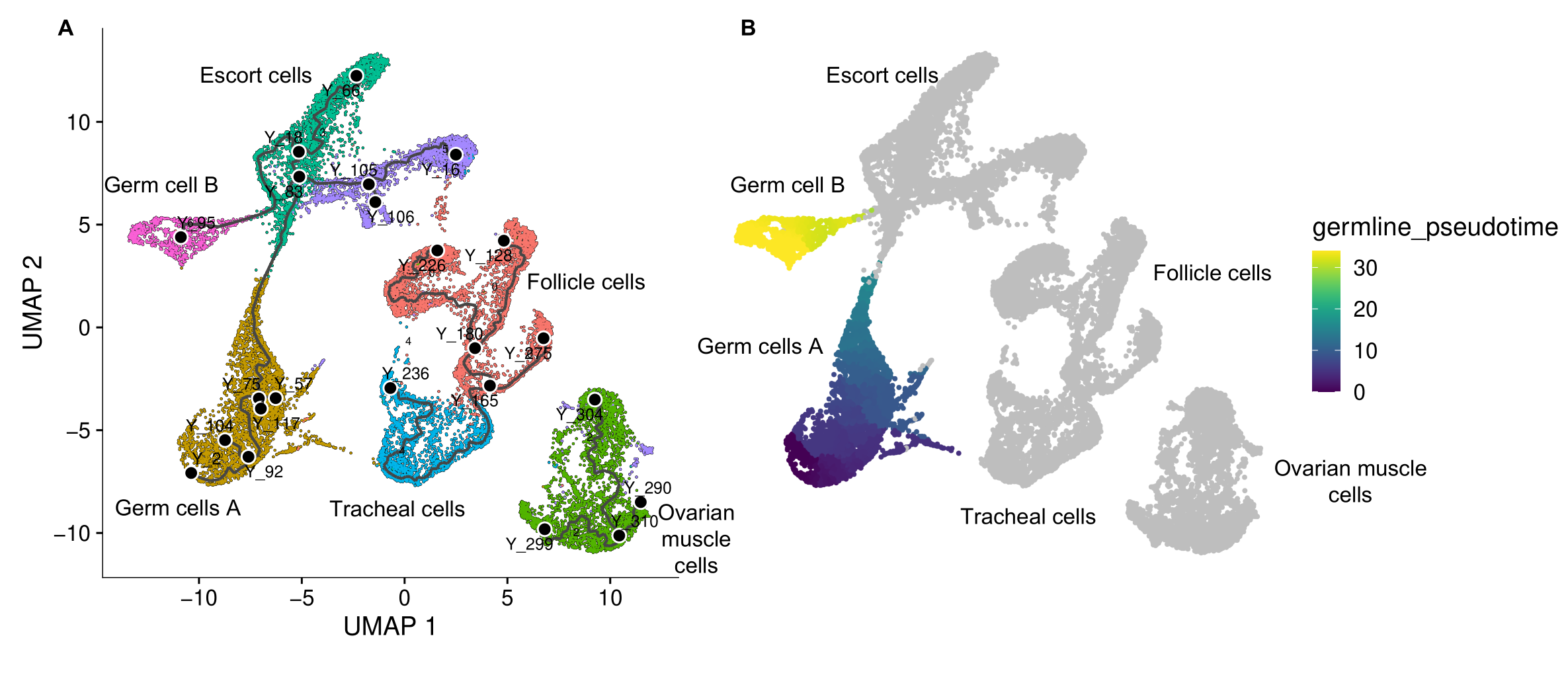

Supplement: S25 Fig — A) UMAP depicting the Monocle3 pseudotime trajectory of all the clusters (all replicates). B) germline pseudotime (Germ cells A and Germ cells B). Y_2 (panel A) was used as the principal node. (TIFF) [file pgen.1011376.s025.tiff]

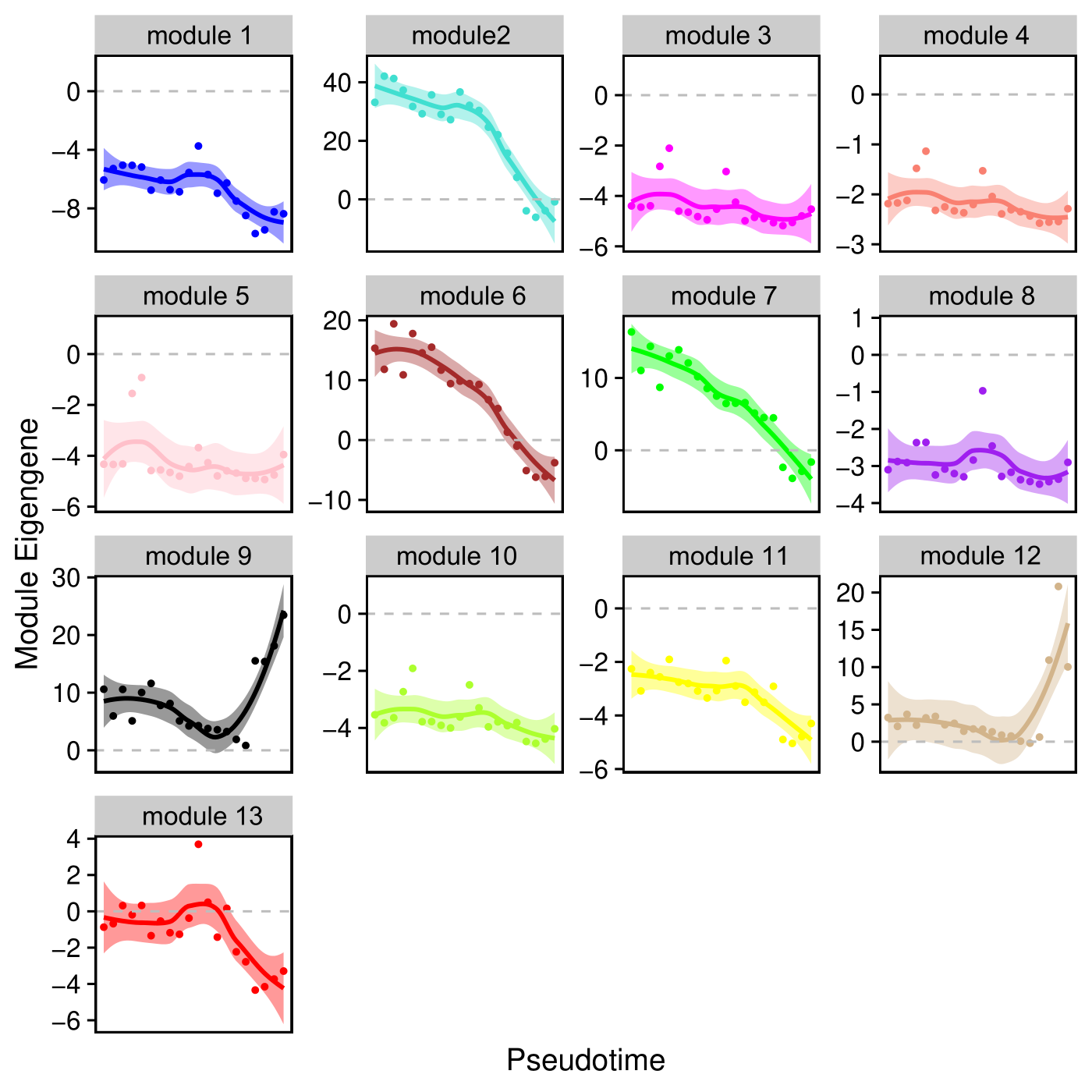

Supplement: S26 Fig — (TIFF) [file pgen.1011376.s026.tiff]
