# Supplementary material for: Single-nucleus atlas of the Artemia female reproductive system suggests germline repression of the Z chromosome
Source: PLoS Genet. 2024 Aug 30;20(8):e1011376. doi: 10.1371/journal.pgen.1011376 (PMC11392275; doi:10.1371/journal.pgen.1011376)
Supplement: Attachment — Submitted filename: Response_to_reviewers (05-07-2024).pdf [file pgen.1011376.s029.pdf]

Dear Dr Elkrewi,

Thank you very much for submitting your Research Article entitled 'Single-nucleus atlas of the Artemia female reproductive system suggests germline repression of the Z chromosome' to PLOS Genetics.

The manuscript was fully evaluated at the editorial level and by independent peer reviewers. Three experts have provided their review comments. While the reviewers agree the work offers novel insights into MSCI, they have raised several technical concerns that, once addressed, should strengthen the manuscript. Based on the reviews, we will not be able to accept this version of the manuscript, but we would be willing to review a much-revised version. We cannot, of course, promise publication at that time.

Should you decide to revise the manuscript for further consideration here, your revisions should address the specific points made by each reviewer. We will also require a detailed list of your responses to the review comments and a description of the changes you have made in the manuscript.

If you decide to revise the manuscript for further consideration at PLOS Genetics, please aim to resubmit within the next 60 days, unless it will take extra time to address the concerns of the reviewers, in which case we would appreciate an expected resubmission date by email to plosgenetics@plos.org.

If present, accompanying reviewer attachments are included with this email; please notify the journal office if any appear to be missing. They will also be available for download from the link below. You can use this link to log into the system when you are ready to submit a revised version, having first consulted our Submission Checklist.

To enhance the reproducibility of your results, we recommend that you deposit your laboratory protocols in protocols.io, where a protocol can be assigned its own identifier (DOI) such that it can be cited independently in the future. Additionally, PLOS ONE offers an option to publish peer-reviewed clinical study protocols. Read more information on sharing protocols at https://plos.org/protocols?utm_medium=editorial-email&utm_source=authorletters&utm_campaign=protocols

Please be aware that our data availability policy requires that all numerical data underlying graphs or summary statistics are included with the submission, and you will need to provide this upon resubmission if not already present. In addition, we do not permit the inclusion of phrases such as "data not shown" or "unpublished results" in manuscripts. All points should be backed up by data provided with the submission.

While revising your submission, please upload your figure files to the Preflight Analysis and Conversion Engine (PACE) digital diagnostic tool. PACE helps ensure that figures meet PLOS requirements. To use PACE, you must first register as a user. Then, login and navigate to the

UPLOAD tab, where you will find detailed instructions on how to use the tool. If you encounter any issues or have any questions when using PACE, please email us at figures@plos.org.

PLOS has incorporated Similarity Check, powered by iThenticate, into its journal-wide submission system in order to screen submitted content for originality before publication. Each PLOS journal undertakes screening on a proportion of submitted articles. You will be contacted if needed following the screening process.

To resubmit, use the link below and 'Revise Submission' in the 'Submissions Needing Revision' folder.

We are sorry that we cannot be more positive about your manuscript at this stage. Please do not hesitate to contact us if you have any concerns or questions.

Yours sincerely,

Li Zhao
Guest Editor
PLOS Genetics

Kelly Dyer
Section Editor
PLOS Genetics

Thank you for your consideration. Please find our responses to the reviewers' suggestions below.

Reviewer's Responses to Questions

Comments to the Authors:
Please note here if the review is uploaded as an attachment.

Reviewer #1: The authors performed single-cell RNA-seq and ATAC-seq in ovaries of the brine shrimp Artemia franciscana, a species with ZW sex chromosomes. Using the well-annotated cell atlas of Drosophila melanogaster, they show that reproductive cell types are well conserved across arthropods. Although the Z chromosome appears to be dosage compensated in somatic cells, where it has approximately equal expression as the autosomes, its expression is downregulated in germline cells. The reduced Z expression is consistent with a lack of dosage compensation in the germline and may also reflect sex-chromosome repression analogous to the MSCI that occurs in mammals.

Overall, I find this to be a well-written manuscript with clear results. It adds to our knowledge of reproductive system organization and sex chromosome gene expression using state-of-the-art methods in a non-model arthropod. Thus, it is a valuable contribution to the field, which consists

mainly of studies in Drosophila and, to a lesser extent, Lepidoptera and Daphnia. The only part of the manuscript that I found difficult to understand was the section "Downregulation of the Z chromosome in the germline", which I will comment on below.

Thank you for your enthusiasm and suggestions for improvements, which we implement below.

1. I am confused by the classification scheme presented in lines 295-300. After going over the 3 bullet points multiple times, I think there are several mistakes:

a) The first bullet point uses "log2(S0/Auto)", while the others use "S0/Auto". I assume it should be log2 in all cases, since some of the values given are negative.

b) In bullet point 2, the "and" statement doesn't make sense, because both percentile cutoffs are based on S0/Auto. Should the second part be "somatic/2".

c) Also in bullet point 2, should the first value be "<= -0.91", which would match the value in the first bullet point?

Thank you for the comment. As you pointed out, the classification scheme had some typos, which we have now fixed. We have also adopted your suggestion to use the arbitrary criteria for the main text, and we have moved the figure with the percentiles' approach to the supplementary material and added the description of the approach in the methods section. We have also generated a separate figure (Supplementary figure 14) for the bootstrapping procedure (the non-overlapping autosomal windows approach that we had originally used).

We added the following to results section:

"In order to check if any cells have expression patterns consistent with additional repression of the S0, we classified all cells in each cluster into bins of decreasing S0:A expression ratio that should reflect the presence of dosage compensation and/or repression of the S0 (S0/Auto ratio for all cells is adjusted by adding 1-median(S0/Auto of somatic clusters)):

- *Complete or partial dosage compensation: S0/Auto > 0.66*
- *Lack of dosage compensation: S0/Auto <= 0.66 and S0/Auto > 0.33.*

- *Repressed: S0/Auto <= 0.33."*

We have also added this sentence to the results:

"As an additional measure, we used percentile-based cutoffs to control for the heterogeneity of Z chromosome regulation status caused by noise, and we recovered the same enrichment patterns (See methods section "Z-chromosome regulation status using percentile-based cutoffs" and Supplementary figure 15)."

And the following section to the methods:

"Z-chromosome regulation status using percentile-based cutoffs

As the within cluster variation in the status of Z-chromosome expression is possibly driven by noise, we implemented more conservative thresholds that apply a 5% false positive rate to the first category in each comparison to provide a noise-sensitive estimate of the cluster-specific enrichments (Supplementary Figure 15):

- *Complete or Partial dosage compensation: S0/Auto > 5th percentile of S0/Auto in somatic clusters (>0.57).*
- *Lack of dosage compensation: S0/Auto <= 5th percentile of S0/Auto in somatic clusters and S0/Auto > (5th percentile of S0/Auto in somatic clusters)/2 (<=0.57 and >0.28).*
- *Repressed: log2(S0/Auto) <= (5th percentile of S0/Auto)in somatic clusters)/2 (<=0.28)."*

2. If I now understand the classification scheme as corrected above, it is rather conservative in classifying "Lack of DC" relative to "Complete DC", and "Repressed" relative to "Lack of DC". This is because the authors use an approach that essentially applies a 5% false positive rate to the first category in each comparison. I understand that this is a conservative approach and should not have a large effect on the statistical analysis that compares proportions between cell types with a chi-square test (although might reduce power in cases where there are a small number of cells in a category). However, it may give misleading results in terms of the percentage of cells in each category as given in lines 303-307. I don't think the authors need to change the way the results are presented in the text/figures, but they should acknowledge in the text that their approach will underestimate the percentage of genes in the "Lack of DC" and "Repressed" categories. Maybe they could add a supplemental figure similar to figure 5, but using more relaxed (albeit arbitrary) definitions that don't rely on outliers to an empirical distribution. For example (on a non-log2 scale),
Complete DC: S0/Auto > 0.66

Lack of DC: S0/Auto < 0.66 and S0/Auto > 0.33
Repressed: S0/Auto < 0.33

We have now used the suggested thresholds in Figure 5. Below are the differences between the percentiles approach and the arbitrary approach. We do see that the percentiles approach underestimates the percentage of repressed cells and cells that lack dosage compensation. However, in both cases, the enrichment of germ cells B for cells lacking dosage compensated and repressed cells remains significant.

Review table 1: Percentage of cells in the different categories of Z chromosomes expression using the two different approaches: arbitrary and percentiles approach.

	Arbitrary approach		Percentiles approach	
	Germ cells A	Germ cells B	Germ cells A	Germ cells B
%Complete or partial DC	66.037212	27.97619	76.534296	42.97619
%Lack of DC	27.825604	55.238095	19.383505	45.833333
%Repressed	6.137184	16.785714	4.082199	11.190476

Below you can find the figure generated using the arbitrary approach (now Figure 5 B and C):

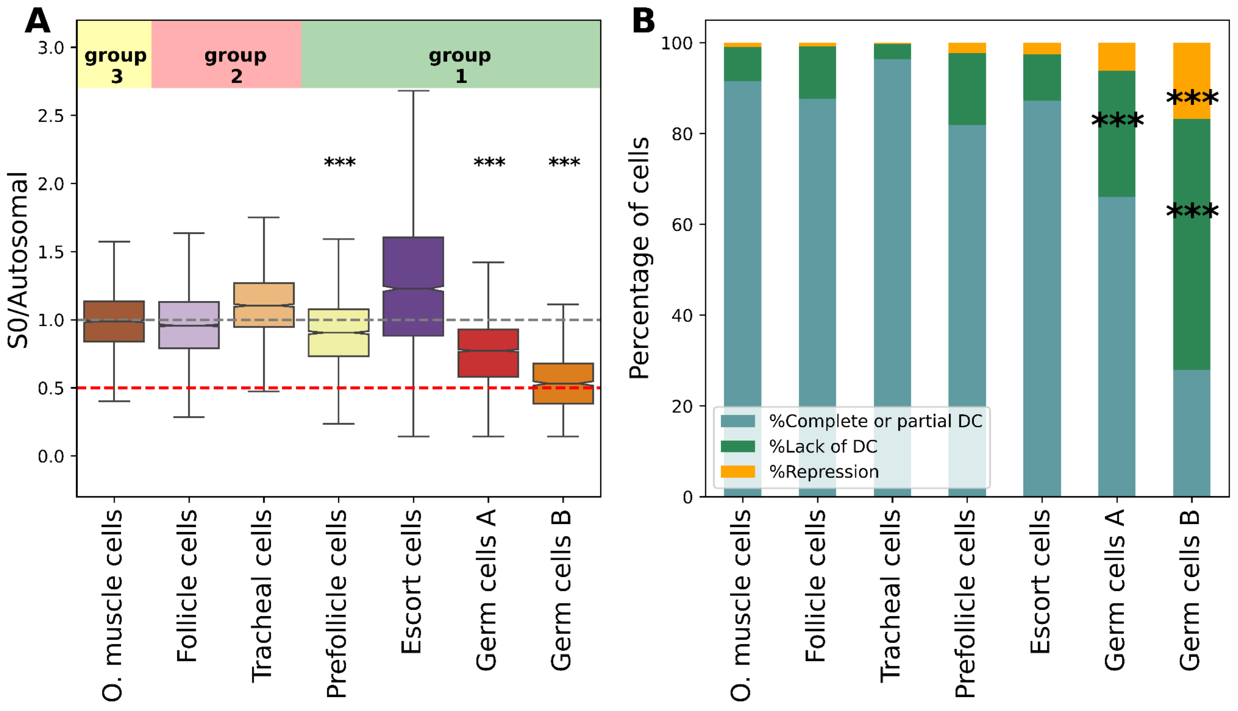

Review figure 1: A) The mean(S0)/mean(Autosomes) expression per cell estimated using the normalized counts matrix. The stars show the significance values for group 3 and somatic clusters comparisons (wilcoxon rank-sum test). C) The percentage of cells that are dosage compensated (DC), lack dosage compensation (Lack of DC) and repressed (Repression). The stars show the significance values comparing group 3 clusters and somatic clusters (%Lack dosage compensation vs rest, and %Repression vs rest using Chi-square contingency test). For the stars, *** denotes p-value <= 0.001, ** denotes p-value <= 0.01, and * denotes p-value <=0.05..

Below you can find the figure generated using the percentiles approach (Panel B is now Supplementary Figure 15):

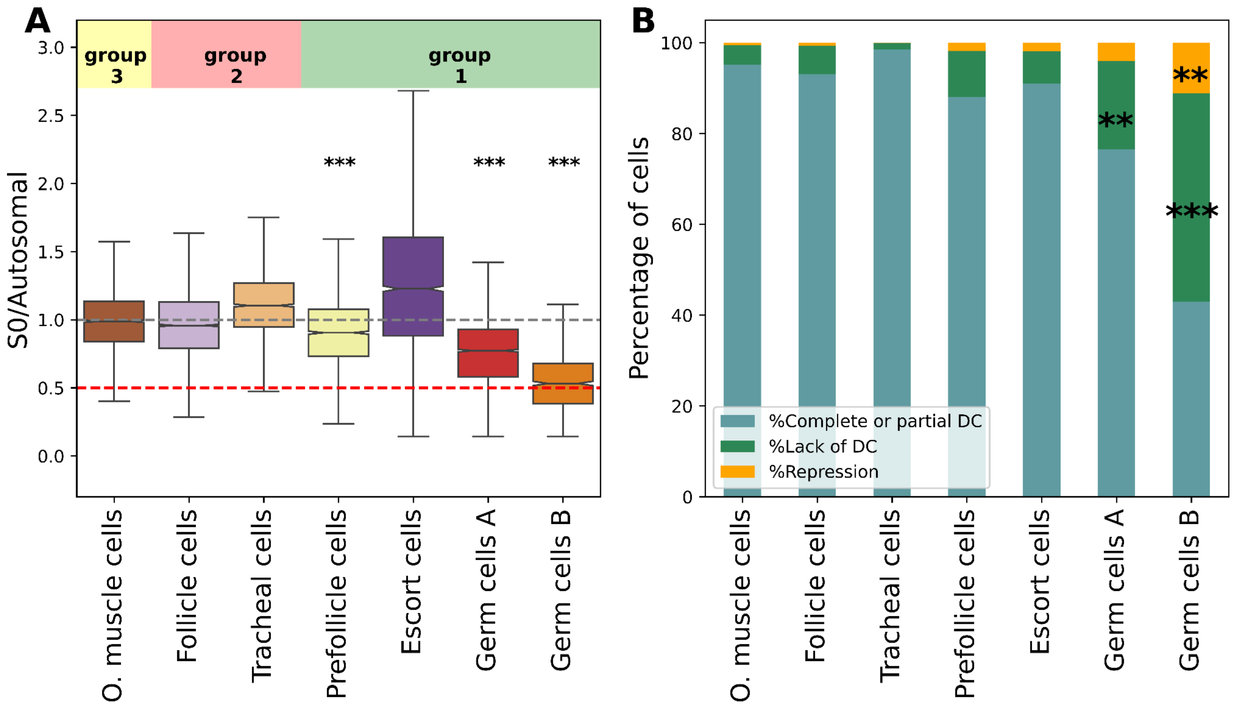

Review figure 2: A) The mean(S0)/mean(Autosomes) expression per cell estimated using the normalized counts matrix. The stars show the significance values for group 3 and somatic clusters comparisons (wilcoxon rank-sum test). C) The percentage of cells that are dosage compensated (DC), lack dosage compensation (Lack of DC) and repressed (Repression) using percentile-based cutoffs. The stars show the significance values comparing group 1 clusters and somatic clusters (%Lack dosage compensation vs rest, and %Repression vs rest using Chi-square contingency test). For the stars, *** denotes p-value <= 0.001, ** denotes p-value <= 0.01, and * denotes p-value <=0.05.

3. The above classification is also confusing, because the authors consider a log2(S0/Auto) > -0.91 as "complete" dosage compensation. However, at the low end this means the X has only slightly higher than half the expression of the autosomes. It might be better to consider this "Complete or partial DC".

We agree that “Complete or partial DC” is a better description, and we now use that throughout the paper.

Minor points

line 20: "Melanogaster" should not be capitalized

Thank you for the comment. We fixed this in the text.

line 87: I don't think "Meiotic" needs to be capitalized here. Similarly, "Meiosis" does not need to be capitalized in line 181.

We fixed both in the text.

line 198: here "drosophila" should be capitalized and in italics

Fixed in the text.

line 289: "The median log2 of the expression ratio in Germ cells B is around -1, which is overall more consistent with lack of dosage compensation than with true Z inactivation". This observation does match the expectation based on gene dose, however, previous studies in Drosophila and birds have observed that the ratio is not as extreme in the absence of DC. It appears that the X (or Z)/Auto is closer to -0.6 (e.g. Itoh et al. 2007; Ellegren et al. 2007; Meiklejohn et al. 2011; Argyridou and Parsch 2018). This would be expected if some genes had gene-specific regulatory mechanisms that balanced their expression. Many genes are part of regulatory networks that have feedback loops to maintain appropriate expression levels. Thus, I find it a bit surprising that Artemia seems to lack this type of regulation and expression appears to be almost entirely controlled by gene dose? If one assumes an expected value of -0.6 for lack of DC, then the observation of -1 might suggest a combination of lack of DC and sex chromosome repression.

We agree with your remarks, and we have added the following sentences to the discussion:

"Additionally, lack of dosage compensation in other species seems to result in less than 2 fold decrease in the expression of X/Z-linked genes (~1.5 in the Drosophila testes and ~1.6 in the chicken gonads) (1–4). In our analysis, the distribution of S0/Autosome ratios per cell in the germ cells B cluster is centered at 0.5 (2-fold decrease in expression). If one assumes an expected value of 0.66 for lack of DC, then the observation of 0.5 might suggest a combination of lack of DC and sex chromosome repression."

Reviewer #2: Overview:
Elkrewi and Vicoso generated the snRNA-seq and snATAC-seq for Artemia ovaries. Uniquely, Artemia has the ZW sex chromosome system which provides the opportunity to determine whether there is MSCI of the Z. Despite little being known about the regulatory programs of Artemia germline, the authors used a myriad of cutting edge SC methods to establish cell types based on similarities to the Drosophila ovaries, including two germ cell populations (A and B). While the expression genes in the S0 region (differentiated and presumably non-recombining)of

the Z decreases (relative to autosomes) in the germ cell populations, the reduction is within the range of loss of dosage compensation instead of MSCI. Interestingly, the authors found a reduced accessibility of the PAR, despite DC being unnecessary, suggesting a chromosome-wide silencing/downregulation mechanism consistent with MSCI.

Comments:
As the authors described, the status of MSCI has been highly contested in several organisms (flies and chicken), despite being clear in others (C. elegans and eutherian mammals). The ZW sex chromosome system of Artemia is a great system to examine this enigmatic germline regulation. The absence of well understood expression of ovarian genes makes cell cluster identity classification particularly challenging. But the authors used several sophisticated approaches to map the cell clusters to the melanogaster ovarian atlas, revealing that despite the great evolutionary distance there is enough similarity in gene expression programs of cell types to enable cell type assignment. I really appreciate the lengths to which the authors took to ensure proper inference of cell types.
My primary concerns pertain to how X:A ratio is inferred and how to interpret reduced expression in relation to the dosage compensation status and how accessibility on the Z is interpreted, especially across stratas.

Thank you for the helpful comments and suggestions. As you will see below, we have improved the inference of the Z:A ratio, and clarified the interpretation of the results.

Primary concerns:
I have a hard time understanding how the authors estimated the Z:A ratio using autosomal “bins”. For each cell they first averaged across the 446 genes in the S0 region. But I do not understand how the denominator (autosomal gene expression) is generated in “bins with the same number of genes across the whole genome”. Since comparisons of Z:A ratio is a key part of the study, the authors must provide a better explanation of their procedure. In addition, what would the distributions look like with a simple ratio of S0 median to autosomal median?

Thank you for the comment. We are now simply plotting the distribution of mean(S0)/mean(All autosomal genes) in the main figure. We use the mean, as medians with single cell data tend to be 0s due to the high dropout rate. We moved the binning approach (which we use essentially as a bootstrapping procedure) to generate an additional measure of confidence for our S0/Auto cluster medians (Supplementary Figure 14). For that approach, we divided each chromosome to non-overlapping sliding windows with the same number of genes as the S0 (446 genes), instead of random sampling of 446 autosomal genes, and we repeated the estimates of S0/Auto for all the windows. The procedure is now described in the methods section:

*“**Estimation of S0/Autosomal ratio using non-overlapping autosomal windows***

To ensure that our S0/Autosomal expression estimates are not affected by the low expression throughout the genome, as is the case for some germline cells (see results section 3), we divided the genome into 48 non-overlapping sliding windows with the same number of genes as

the S0 (446 genes). We reasoned that regions of similar gene counts as the S0 are as susceptible to the low detection rates and non-biological zeros that affect single-cell RNAseq data, and can therefore be used to ensure the overall patterns are not technical artifacts. In supplementary figure 14, we show the distribution of the per cluster medians of S0/Autosomal window for all the 48 windows."

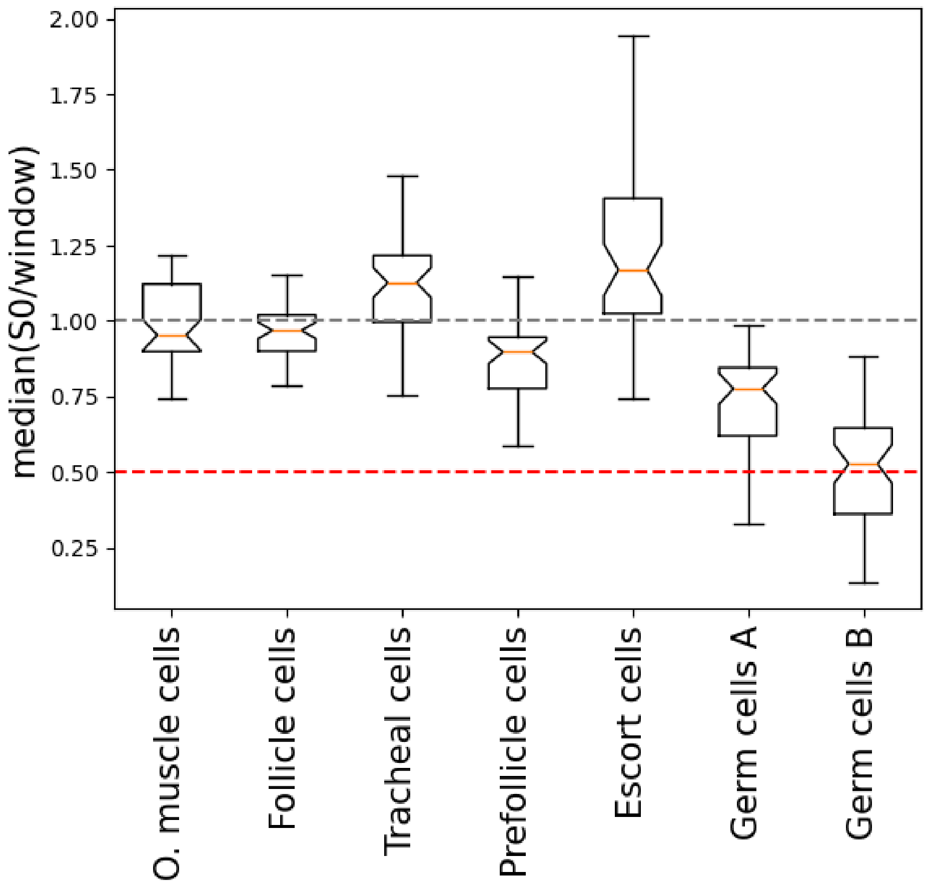

Supplementary Figure 14: Cluster medians of mean(S0)/mean(window) per cell) estimated using 48 non-overlapping genomic windows with the same number of genes as the S0.

Please note that these different approaches to analyze the data give qualitatively similar results, supporting the robustness of our conclusions.

The authors divided the germline cells into those showing complete dosage compensation (of S0), lack of dosage compensation, and repressed. Cells are considered to have "complete dosage compensation" if their log2(S0/Auto) is >-0.91. This equates to a lower-bound ratio of 0.532 which is typically considered no dosage compensation. The lack of dosage compensation is categorized by the cutoff of "<=0.91 and >-1.91" which equates to upperbound and lowerbound of 1.879 and 0.266, respectively which is nonsensical (Did the authors mean <=

-0.91). The repressed category has a cutoff of <= -1.91 equating to a lower-bound ratio 0.266. I am not sure if there are typos, but these expression range classifiers are strange. There are also instances of switching from log scale to linear scale. E.g. “S0/Auto in somatic clusters -1”, do they mean log(S0/Auto) - 1?

Thanks for the comment and we apologize for the confusion caused by the typos. As you pointed out, our original thresholds were:

- The lack of dosage compensation cutoff was log2(S0/Auto) <=-0.91 and >-1.91, which would be S0/Auto <=0.53 and >0.266.
- The repressed category cutoff was log2(S0/Auto) <= -1.91 or S0/Auto <0.266.

Based on the comments of reviewer 1 and yours (see also our response to them above), for the main text (Figure 5), we now use:

- The lack of dosage compensation cutoff is S0/Auto <=0.66 and >0.33.
- The repressed category cutoff is S0/Auto <= 0.33.

For the Supplementary Figure 15, we used the more conservative percentile based cutoffs:

- The lack of dosage compensation cutoff is S0/Auto <=0.57 and >0.28.
- The repressed category cutoff is S0/Auto <0.28.

While specific percentages necessarily change, this does not affect our conclusions (Germ cells A seem to lack dosage compensation, Germ cells B lack dosage compensation but a subset also seems to show stronger repression of the Z).

Odd cutoffs aside, classification of cell types like this implies that cells within the same cluster can have different Z chromosome regulation status. While this could be the case, alternatively, the cell-by-cell variation in Z:A ratio could just reflect a noisy distribution (especially when overall readcount/expression is low as is the case for germ cells B) and the median/mean is closer to the true state of the cell population. A diagnostic way to show that there are indeed different population of cells (some with DC, some no DC, and some repressed/inactivated) in germ cells B would be a clear multi-modal distribution. A unimodal distribution would suggest that noise is driving the spread and cell type classification.

We agree with your concern, which is why we used the percentile-based cutoffs, i.e. we assume that the heterogeneity of Z chromosome regulation status in the non-germline clusters is caused by noise and therefore can be used as the baseline for comparison with the germline cells. But we agree that it is possible that we observe cells with different Z expression status because of a broad distribution rather than because of an actual biological difference (such as the presence of nurse cells and oocytes in the germline, or germline cells at different timepoints in their developmental trajectory). We have plotted the distributions of mean S0/autosomes for different cell clusters as you suggested. Germ cells A have a median of 0.77 with a shoulder towards lower values, which suggests that some cells are compensated and some are not. On the other hand, the whole distribution of germ cells B is shifted (median = 0.53), suggesting a complete

absence of dosage compensation. Furthermore, there is a more prominent bump on the left side (a similar bump is seen in germ cells A, but to a lesser extent), which might suggest that the repressed cells are indeed a distinct group of cells. While we want to be careful with our interpretations as it is tricky to characterize what is actually happening at a stage of overall reduction in transcriptional activity, these distributions are generally in line with our conclusions.

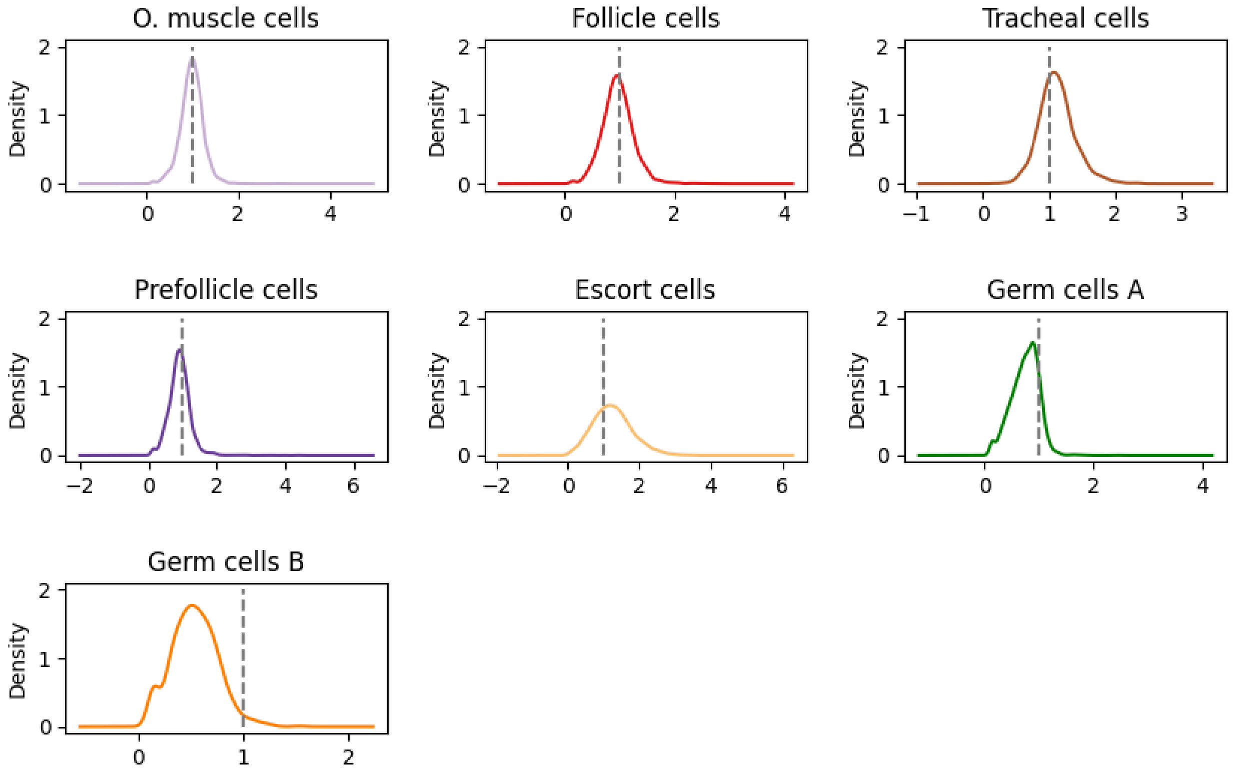

Review figure 3: Distribution of mean(S0)/mean(Auto) values per cell in the different clusters. Dashed-lines are at 1.

In addition, it is also worth noting, if the Z-repressed cells indeed represent a distinct group of cells, the number of cells undergoing MSCI (or more accurately MSCRepression) appear to be a very small population of the germ cells.

We agree, and we now clearly say that it is a small subset of cells:

"While these results point to a ***small*** *subset of germline cells* ***(less than 20% of the cells in the cluster)*** *showing at least partial Z-inactivation, it is notoriously difficult to fully exclude that absence of dosage compensation, along with sparse and noisy data, could create such a pattern."*

The authors wisely looked at the PAR and younger stratas on the Y to infer MSCI. Loss of dosage compensation and MSCI should manifest in different patterns. The significant decrease of PAR expression is perhaps the most convincing evidence of repression to me, since it doesn't need dosage compensation. As such, I would recommend the authors display Figure S12 as a main figure (and perhaps a schematic breakdown of the Z chromosome architecture based on their earlier study). However, there are other oddities that make it hard to interpret the PAR expression, including significantly reduced expression across many somatic cells, with prefolicle being significant. What would explain the unexpected downregulation in a somatic cell type? How many genes are on the PAR?

Thank you for the remark. The pseudoautosomal region in our annotation has around 1378 genes. We have added the number of genes in the different regions to Supplementary Figure 16 and a schematic of the Z-chromosome. As the figures in the manuscript are quite dense already, we decided to leave this one in the supplementary, but we would be happy to add a panel to Figure 5 if you think that is more suitable.

We have made an additional figure, where the PAR/Auto values were not adjusted by the median of somatic clusters, and where the different clusters were split by their Z expression classification. Review Figure 4 (below) shows that the distribution of PAR/Auto values for the majority of clusters is centered at around 1 (median PAR/Auto is 1.1 for Follicle cells, 0.987 for Tracheal cells, 1.076 for Prefollicle cells). Muscle cells seem to have higher expression of the PAR region compared to the other cell types (median PAR/Auto =1.457), which is why even prefollicle cells are significant when muscle cells are included in the somatic controls. What is worth noting is that even the repressed cells in the non-germline clusters have median(PAR/Auto per cell) around 1 (with the exception of the repressed category in Tracheal cells, which has 6 cells only). This suggests that their classification is likely due to noise and not to an actual reduction of the expression of their Z chromosome. The germ cell clusters, on the other hand, seem to have PAR/Auto values consistent with their S0/Auto expression.

Despite all of that, we do agree that prefollicle cells seem to show many similarities with the germline cells, which is consistent with their assignment to group 1 based the dendrogram and heatmap of the correlation matrix of the mean expression values per cluster (Supplementary Figure 5). We mention this in the results (L183-189) and (L416-419) in the discussion.

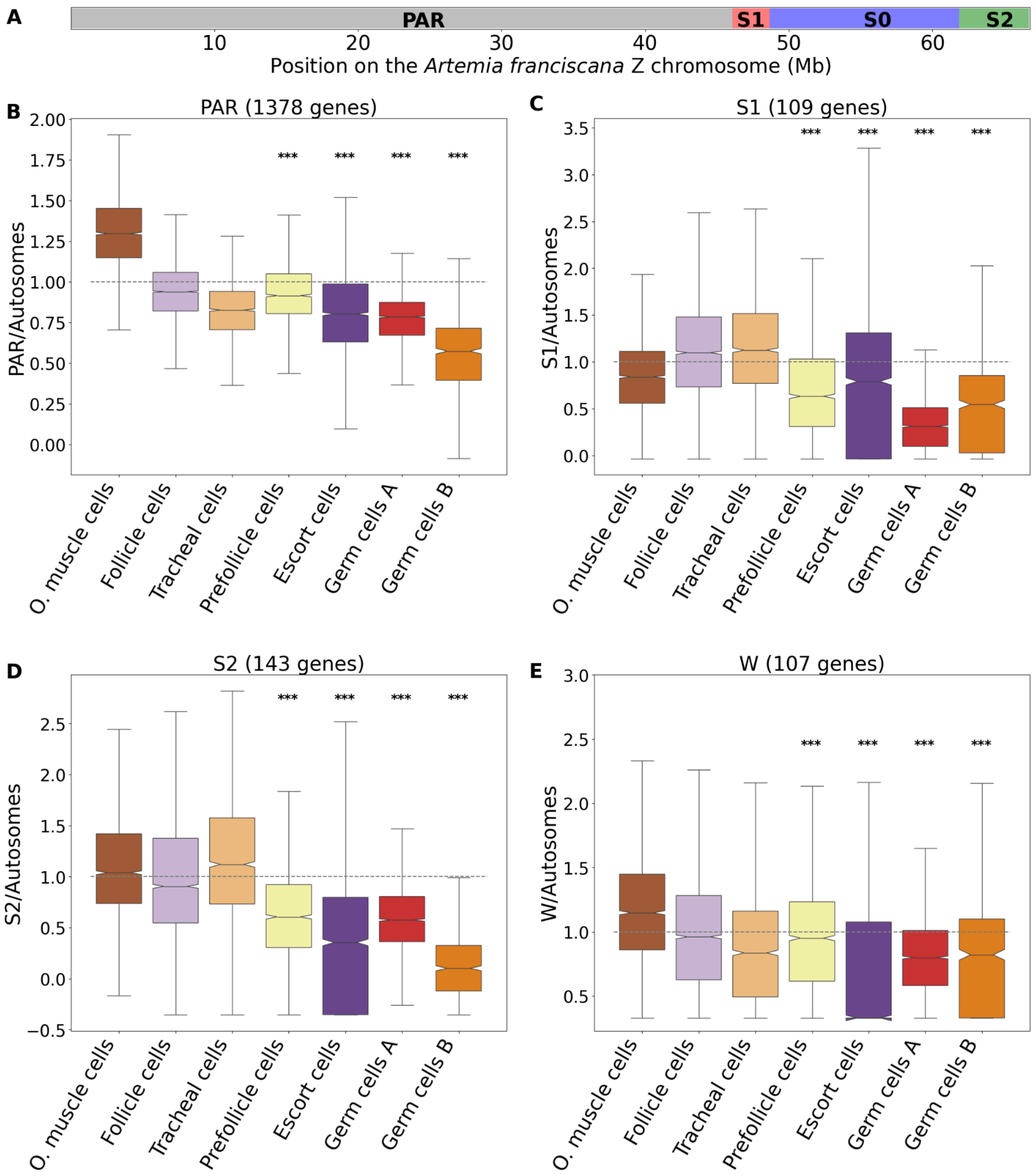

Supplementary figure 16: A) The structure of the Z chromosome as described in (Bett et al., 2024), with the large pseudoautosomal region (PAR), the differentiation region (S0), and two younger strata (S1 and S2). B) PAR/Autosomes expression per cell C) S1/Autosomes expression per cell D) S2/Autosomes expression per cell E) W/Autosomes expression per cell. The normalized counts matrix was used for all the estimates.

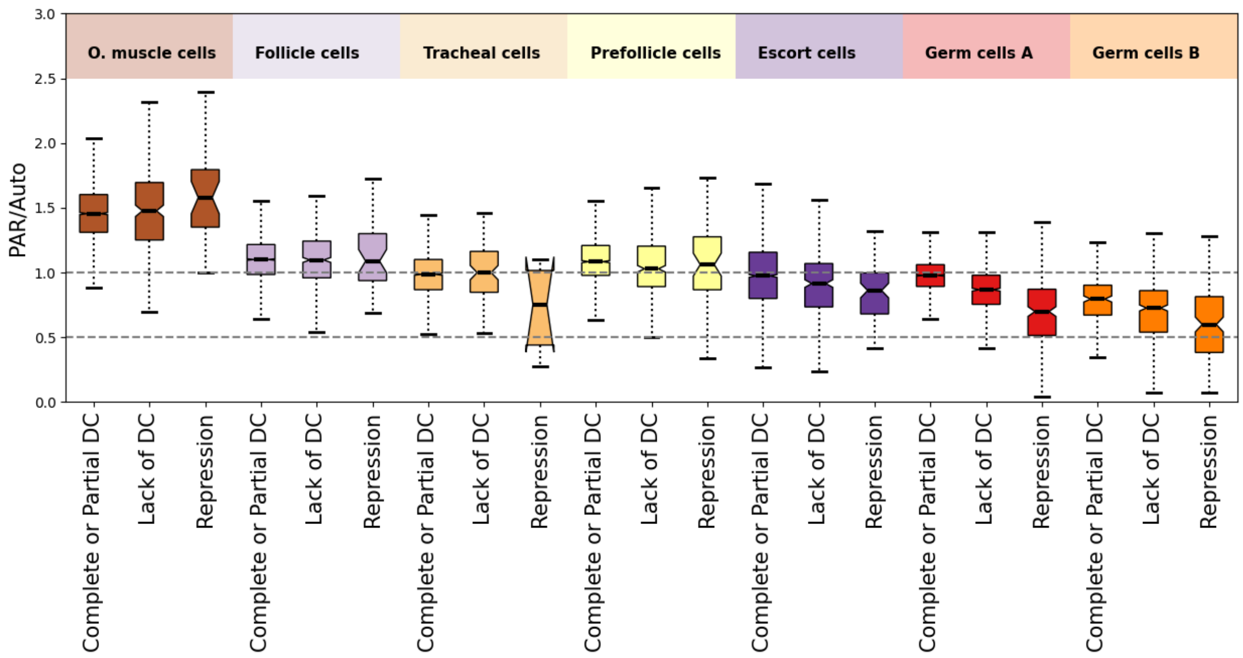

Review Figure 4: The mean(PAR)/mean(Autosomes) expression per cell estimated using the normalized counts matrix. Clusters were split based on the Z-expression status.

Using sn-ATACseq the authors argue that the S0 is less accessible as support for repression instead of loss of DC. While the reduced accessibility of the S0 is significant and notable, it can also be explained by the copy number difference, i.e. the PAR has two copies while the S0 has one.

Thank you for the comment. We agree that based on copy-number, the expectation is that the S0 should have half the accessibility of the autosomal and pseudoautosomal regions. Therefore, the important comparisons are for the same regions, between the germline and non-germline clusters (e.g. PAR in soma versus PAR in germ cells). This is the reason we include a control label to highlight the controls for each comparison, and we have now added lines to avoid the confusion.

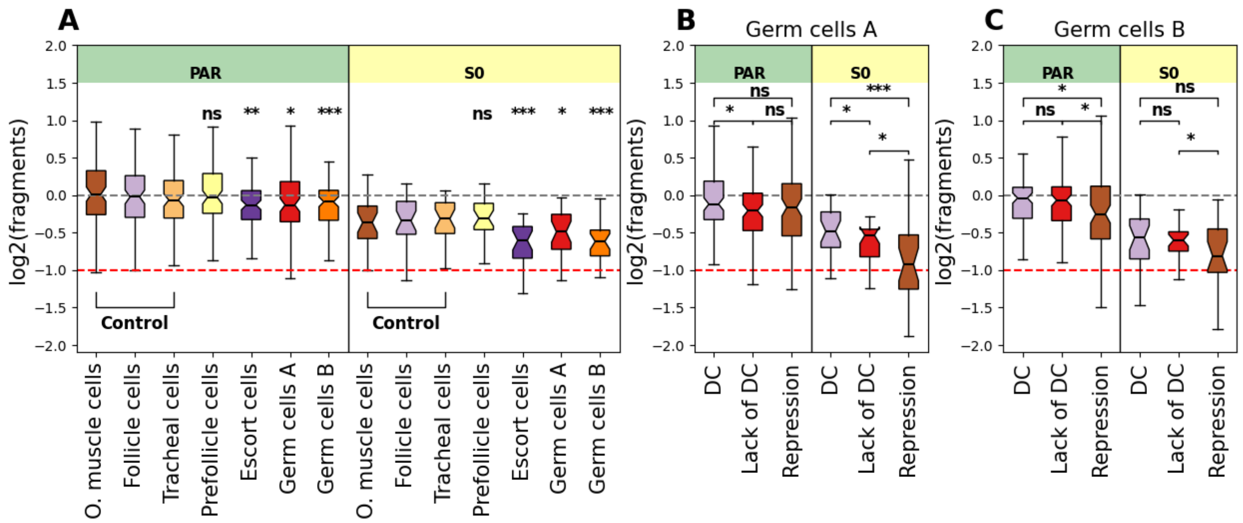

Figure 6: Decreased Z-chromosome accessibility in the germline clusters. A) log2(fragments) for autosomal, PAR, and S0 in windows of 500,000 bp estimated from pseudo-bulks of all nuclei within a cluster adjusted by subtracting the median(log2(autosomal windows)) of each cluster. The stars show the significance values for the comparisons between group 3 clusters and somatic clusters (wilcoxon rank-sum test). B) and C) log2(fragments) for autosomal, PAR, and S0 in windows of 500,000 in Germ cells A and B split into pseudo-bulks based on the expression zone of the Z chromosome adjusted by subtracting the median(log2(autosomal windows)) of each cluster. The stars show the significance values for comparisons between the different categories (wilcoxon rank-sum test). For the stars, *** denotes p-value <= 0.001, ** denotes p-value <= 0.01, and * denotes p-value <=0.05. The red line is at -1, where it signifies a two fold decrease in the number of fragments.

Other concerns/questions/comments:
In the Drosophila testes, Y-linked genes are upregulated in the meiotic stages. This is particularly striking on a recently formed neo-Y chromosome and is thought to result from the masculinization of the male-specific chromosome (perhaps due to sexual conflict). In other testes cell types Y-linked genes are typically lowly expressed. However, the authors observed downregulation of the W in the meiotic stages instead and “dosage-compensated” expression in several of the somatic tissues. This is very counter-intuitive. How many genes are found on the W, and do the authors have an explanation for why they appear dosage compensated and become down regulated?

In mammals, both the X and the Y are repressed during meiosis, but as you point out this is not the case in Drosophila, so it is not completely straightforward to set expectations for the Artemia W. As all cell types have one W chromosome in the female somatic tissues, there is no reason for the W to not have the same overall expression levels across the different cell types. Additionally, as the W is only in females, there is no dosage compensation per say, and the W genes are individually regulated to their optimal level (which may differ across cell types).

We have included 107 W-genes in this analysis (Review Figure 5), based on the W scaffolds described in (5). The majority of those genes seem to be lowly expressed overall. However,

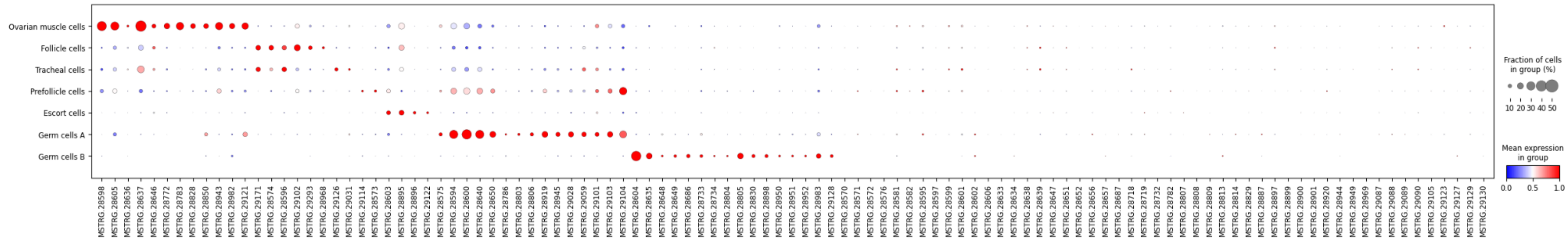

some of them seem to have germline specific expression patterns, consistent with a minor feminization of the W.

Review figure 5: Expression patterns of the W genes on the putative W-scaffolds from (5).

At several points in the manuscript, the authors state/imply there is no dosage compensation in the Drosophila male germline and cite several papers such as Witt et al 2021. Recent genomic and scRNA-seq studies have pretty clearly demonstrated the presence of DC during at least the early stages of the male germline, including Witt et al 2021, Mahadevaraju 2021, Anderson et al 2023, Wei et al 2024, and others.

Thank you for the comment, we have replaced “in the male germline” with “in spermatocytes” in the following sentence:

“In the case of fruit flies, a recent study showed that the X chromosome of Drosophila melanogaster is not enriched for silencing marks in ***spermatocytes****, suggesting the absence of MSCI (6)”*

In the other instance, we do mention the specific cell types, where the absence of dosage compensation has been reported:

“In Drosophila males, the lack of dosage compensation manifests in the absence of X chromosome upregulation in ***primordial germ cells, spermatocytes and spermatids*** *(4,7,8)”*

Additionally, we now added that we mainly observe an enrichment in cells lacking DC in the meiotic and post-meiotic cells types in the Drosophila testes dataset:

“We used the same approach and thresholds to check whether we see a similar pattern in the Drosophila testes dataset (9), and we only observe an enrichment in cells lacking dosage compensation in some of the germline clusters ***(mainly in the meiotic and post-meiotic cell types),*** *but no cells show extreme repression of the X chromosome (Supplementary figure 20).”*

In Line 74-75. The description of dosage compensation in the female germline of mammals should be better described than merely “disappear/reverse”. It is “reconfigured” and rather complex, including a period of hyper-transcription and then dosage balance via expression of

both Xs.

Thank you for bringing this to our attention, we have now rephrased the sentence as follows:

“This sex chromosome-specific epigenetic regulation seems to disappear/reverse in the germline cells of Drosophila males (7,8) and undergo extensive reconfiguration in mammalian females, with a period of hyper-transcription before reaching dosage balance (X:A ~ 1) (10,11).”

The authors should consider discussing the results from a recent publication investigating germline dosage compensation loss vs MSCI in a Drosophila species with neo-sex chromosomes.

We have now added a sentence discussing the findings from this paper:

“In the case of fruit flies, a recent study showed that the X chromosome of Drosophila melanogaster is not enriched for silencing marks in spermatocytes, suggesting the absence of MSCI (6). Another study used single-cell RNAseq in Drosophila miranda, which has the ancestral Drosophila X (Muller element A), along with two younger X-linked chromosome arms (Muller AD and Muller C), found that all three X chromosomes have expression patterns consistent with a lack of dosage compensation in late spermatocytes and spermatids (12).”

The cell grouping (1,2, and 3) is flipped in Figure 5B.

Thank you for pointing this out. We have now changed the groups in the Figure.

Line 259-260, the authors state “RNA counts decrease across the germline pseudotime”. However Figure 4E shows an increase in expression at the end of the pseudotime.

Yes, we agree that we should have commented on the increase. We added the following sentence to the main text:

“The RNA counts seem to rise towards the end the germline pseudotime, which could be a sign of transient transcriptional reactivation, similar to what happens in Drosophila oocytes during oogenesis, between stages 9 and 11 (prophase I arrest ends at stage 13)(13). However, as the ATAC counts do not show a similar pattern, the pattern may not be biological.”

In Figure 6 What are red dotted lines supposed to represent?

We moved the red line to -1, where it signifies a two fold decrease in the number of fragments, and we added that information to the legend.

Reviewer #3: In this manuscript, the authors performed single nucleus RNA-seq and ATAC-seq of the Artemia female reproductive system. This study presented a comprehensive single nucleus atlas of the adult Artemia franciscana female reproductive system. For example, the study identified different cell clusters, uncovered the gene expression dynamics across different cells, and shed light on the status of MSCI in the ZW system. Together with flycellatlas data, the authors also showed the conservation and divergence of female reproductive system between Artemia and Drosophila. However, I have some concerns before I would recommend the manuscript to be published.

Thank you for the support and for bringing up several aspects of the data that we have now explored in more detail.

1. There are a decent number of cells in the identified Germ cells B that can map to Artefact cells in flycellatlas data. Including these cells might be problematic, as these cells, according to the flycellatlas paper, expressed nearly all genes. The authors should clarify if the Artefact cells were included in subsequent analysis.

This is a very good point. First, we should note that our own data shows no evidence of including artifacts, and we map whole clusters across species, not individual cells. Therefore even if a subset of cells is marked as artifact in Drosophila, this should not invalidate the classification of our own clusters.

That said, we have actually been puzzled by the cells labeled artefact in the Drosophila ovaries, as they cluster with the germline cells and show highly similar expression patterns to these cells (Supplementary figure 14 for a correlation analysis of the original Drosophila data). In the main text of the fly cell atlas paper, they mention the artefact cells in the context of the dataset integrating all tissues, as a cluster where they see the expression of three incompatible markers (grh for epithelial cells, Mhc for muscle cells, and Syt1 for neurons). We do not see any expression of those markers in the artefact cells in the ovary (Review Figure 6).

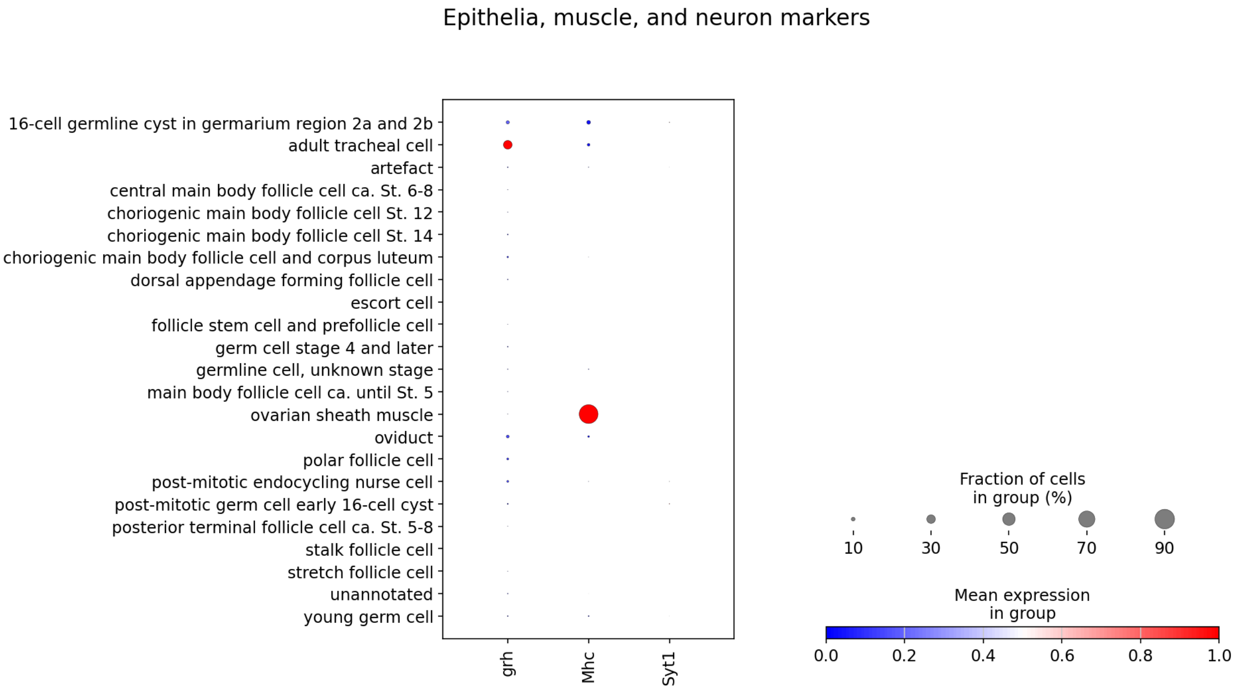

Review figure 6: Dotplot of the Epitelial, muscle and neuron markers in the ovary dataset from the fly cell atlas.

We have also looked at the expression of the markers of artefact cells described in the supplementary material of the fly cell atlas paper (science.abk2432_table s2.xlsx) in the ovarian dataset (listed in Review Table 2 below). The artefact cells do not actually show any enrichment in the expression of these markers compared to the other ovarian cell types (Review Figure 7 A and B).

Review table 2: Artefact cluster markers from the fly cell atlas supplementary material

Tissue	Annotation	Annotation ID (Fbt)	Broad annotation	Curators Markers	Top Computed Markers
body, haltere, head, malpighian_tubule, ovary	artefact	Manual Annotation	artefact	Cp18, Cp38, Fas3, Hml, Ppn, Sh, Vm26Aa, Vm26Ab, Vm32E, bam, dec-1, nos, orb, para, psd, tj, vas	Cp15, Cp36, Cp19, Vm34Ca, Vm26Ab, Cp38, Cp18, Vm26Aa, Cp16, Vml

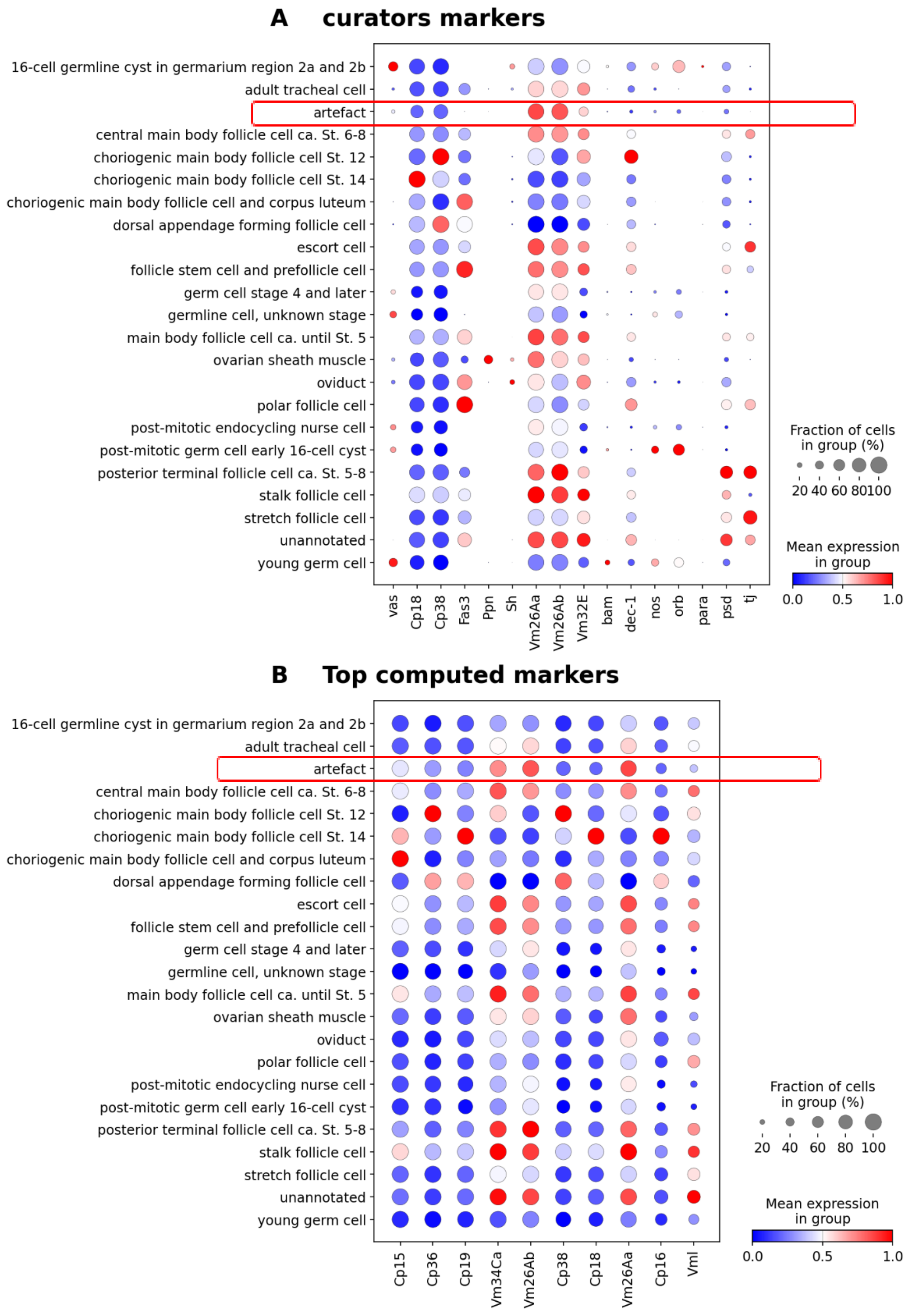

Review figure 7: Dotplot of the artefact cluster “Curators markers” and “Top computed markers” shows no enrichment in the artefact cluster compared to other cell types in the ovary data from the Fly Cell Atlas.
.

Nonetheless, despite our analyses pointing towards the artefact cluster in the *Drosophila* ovary being a real germline cluster, we have repeated the integration excluding it (Review figure 8). While the alignment scores are higher, the major clusters (including germ cells A and B) retained their identity. The prefollicle cells (which had a very low alignment rate in the original analysis), now map to stretch and stalk follicle cells, and the escort cells map better to main body follicle cells. This uncertainty in the assignment of the Prefollicle and escort cells was already highlighted in our results (L183-189). As we believe this Drosophila cluster is real, we prefer to keep our original assignments in the main paper.

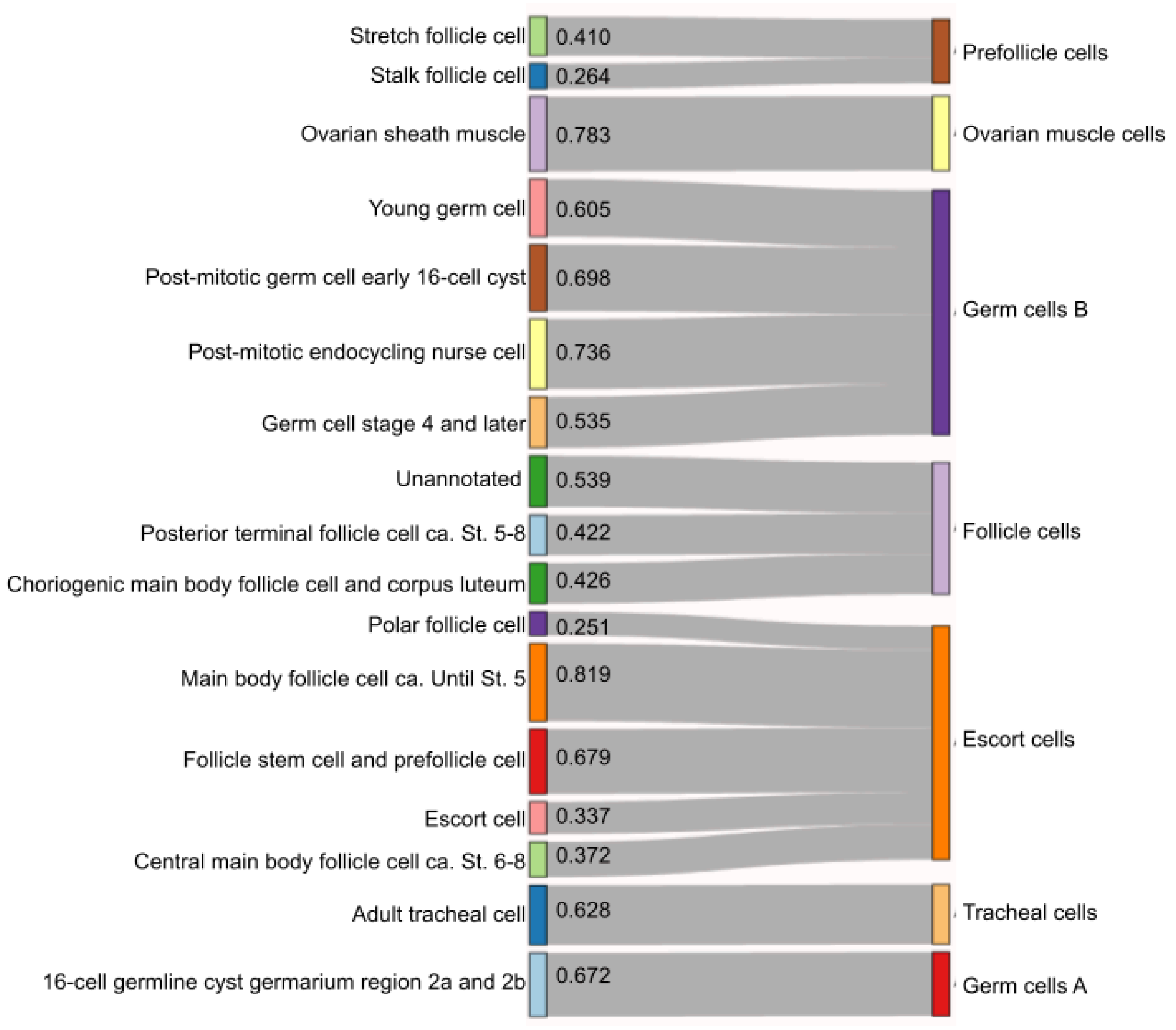

Review Figure 8: Sankey plot showing the mapping between the Artemia and Drosophila clusters (artefact cluster excluded), with the numbers corresponding to the alignment scores between the clusters.

2. In Figure 4A and 4B, the authors concluded that transcription is repressed in late germ cells

by comparing the number of RNA/ATAC counts in different cell clusters. I wonder if the authors used normalized RNA counts for each gene per cell. I assume that the authors used total RNA counts per cell cluster. This may raise concerns, as germ cells B cluster has much fewer number of cells and the comparison is not valid. For example the conclusion in line 399-405 would not stand if the authors used the total counts instead of normalized counts.

We used the nCount_RNA (UMI counts) per cell for the boxplot. A recent paper showed that the 10x genomics UMI counts can be used to estimate transcript content in cells/nuclei, making it possible to detect/compare the global transcriptional output between cell-states (14). We have implemented their approach of absolute scaling (sum(log2(UMI counts +1)), and we recover the same pattern as using the sum(UMI counts) directly.

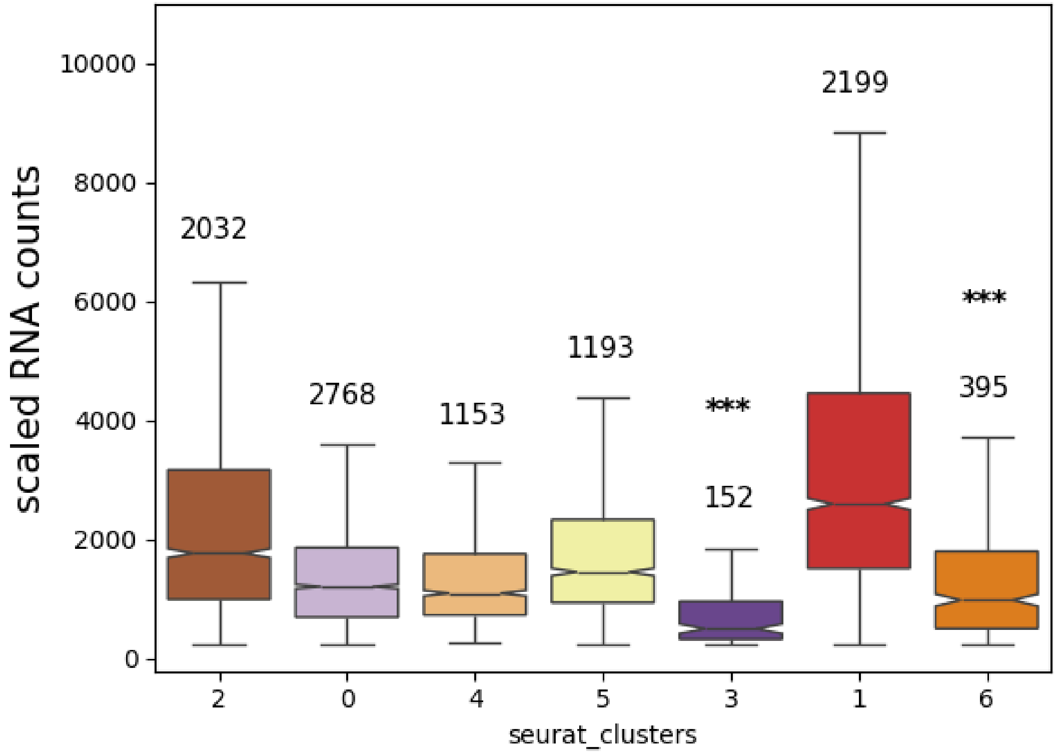

Review Figure 9: The snRNA-seq counts per cell in the different clusters (replicates 3 and 4, number of nuclei above each boxplot).

3. One of the major significances of the work is the discussion of the conservation and divergence between Artemia and Drosophila female reproductive system. In the introduction, the authors described and referred to literatures about the absence of MSCI in the male germline of fruit flies. In the results, the authors showed a clear downregulation of Z chromosome in the germline and repression of transcription in late germ cells. I wonder how much conservation and divergence in this pattern would be expected in Drosophila female germline cells.

This is an interesting point that has, to our knowledge, not been explored. This may be in part because dosage compensation in Drosophila happens in males, and the changes that give rise to MSCI should only affect the heterogametic sex, and therefore should not be observed in the homogametic sex. The expectation may therefore be that no difference in X expression between the germline and the soma is observed in Drosophila.

4. In Figure 6, I am pretty sure the authors used autosomal windows to normalize the data. The authors should adjust the captions or labels in Figure 6 to reflect their analysis.

This is correct. For figure 6, we do report in the legend that we adjust by subtracting the median of autosomal windows: *"log2(fragments) for autosomal, PAR, and S0 in windows of 500,000 bp estimated from pseudo-bulks of all nuclei within a cluster adjusted by subtracting the median(log2(autosomal windows)) of each cluster."*

5. Line 89, the authors described that MSCI in chickens and fruit flies were disputed afterward. However, the authors did not list references for the absence of MSCI in fruit flies in fruit flies.

Thank you for the comment. We now report the two recent references directly after the sentence referring to the disputed results:

"In the case of fruit flies, a recent study showed that the X chromosome of Drosophila melanogaster is not enriched for silencing marks in the male germline, suggesting the absence of MSCI (31). Another study used single-cell RNAseq in Drosophila miranda, which has the ancestral Drosophila X (Muller element A), along with two younger X-linked chromosome arms (Muller AD and Muller C), found that all three X's have expression patterns consistent with the lack of dosage compensation in late spermatocytes and spermatids (32)."

6. In line 277, the authors the expression of the differentiated region of the Z chromosome, to test whether MSCI presents in Artemia. The authors should briefly describe the term "differentiated region" in the main text, as this definition is important for readers to understand the whole idea.

We added a short explanation for S0 in the text:

"After identifying the different cell types in our dataset, we explored the expression of the differentiated region of the Z chromosome (A previously identified ~13 MB Z-linked region with half the coverage in females compared to males, which we refer to as S0 (52)) to assess whether meiotic sex chromosome inactivation is present in Artemia."

7. Line 284, the authors stated that "we estimated the ratio of the mean expression of the genes in the S0 region (446 genes) and of bins with the same number of genes across the whole genome in each cell (Figure 5B shows mean(S0/bin1 +S0/bin2 +..+ S0/binN))". This is unclear, as the axis label of Figure 5B is log2(S0/Autosomal). Can the authors elaborate this?

Thank you for the comment. We have now removed the windows approach from the main figure, as both you and the other reviewers found it confusing, and the legend now reflects the use of mean(S0)/mean(Autosomal genes).

8. Related to the point above, the authors should make clear descriptions as for the metrics used and how they were used in Figures 4-6. The descriptions in the current main text were confusing.

We apologize for the confusion, which the other reviewers also pointed out. As mentioned above, we now stick to simply plotting mean(S0)/mean(autosomes) in the main figures, and hope that these are now easier to interpret.

9. Line 412, the authors checked three genes that play important roles in chromatin remodeling of the Drosophila oocyte. The authors should cite the original work(s). I am also curious if there are other important genes and if these genes are expressed in Drosophila.

In the paper (13), the authors knocked down different chromatin remodelers to see what effect they have on oocyte H3K4me3 and H3K27me3 levels. Out of 46 different knock-downs, three remodelers introduced significant disturbances to the oocyte epigenome: dKDM5 (LID), Ash1 and Bap1. Those are the three remodelers we reported. We realized the reference was missing from line 412, and we have now added it -- thank you for noticing this.

10. In Figure 3D, can the authors elaborate "Module Eigengene"? In Figure 3E and 3F, the authors used strength of enrichment, could the authors elaborate how they performed Go analysis and how the strength was calculated? I would assume that the PPI enrichment p-value were obtained from the STRING database, can the authors clarify?

Yes, the PPI enrichment p-value was obtained from STRING DB (string-db.org), in addition to the GO analysis stats. According to the website, strength is calculated as the Log10(observed/expected), which is the ratio between the number of proteins in the network that are annotated with a term and the number of proteins that are expected to be annotated with this term in a random network of the same size.

We now mention explicitly in the methods section (Protein Interaction network and GO enrichment analysis) that the GO analysis was also performed using StringDB:

"and the translated sequences were uploaded to https://string-db.org/, where the PPI and GO enrichment analyses for the modules were performed."

We have also added an description of Strength to the legend of Figure 3:

"Strength (retrieved from StringDB) is estimated as the log10(number of observed proteins with a term/ number of expected proteins with the term in a random network of the same size)."

11. Related to the point above, in line 219, the authors stated that “3 had significant enrichment”. Does this mean significant PPI enrichment or “Go analysis” enrichment? As for the 3 modules, I guess they are module 6, 7, and 9 since the authors showed the enriched biological processes of the three modules. Other than the three modules, module 2 (germ cells A specific) and 12 (germ cells B specific) seem to be significant too. The authors should clarify why the two modules are ignored in further analysis.

We only included the modules with significant PPI enrichment only. Below are the figures for module 2 (1363 genes) and module 12 (61 genes), which we have now added to the supplementary material:

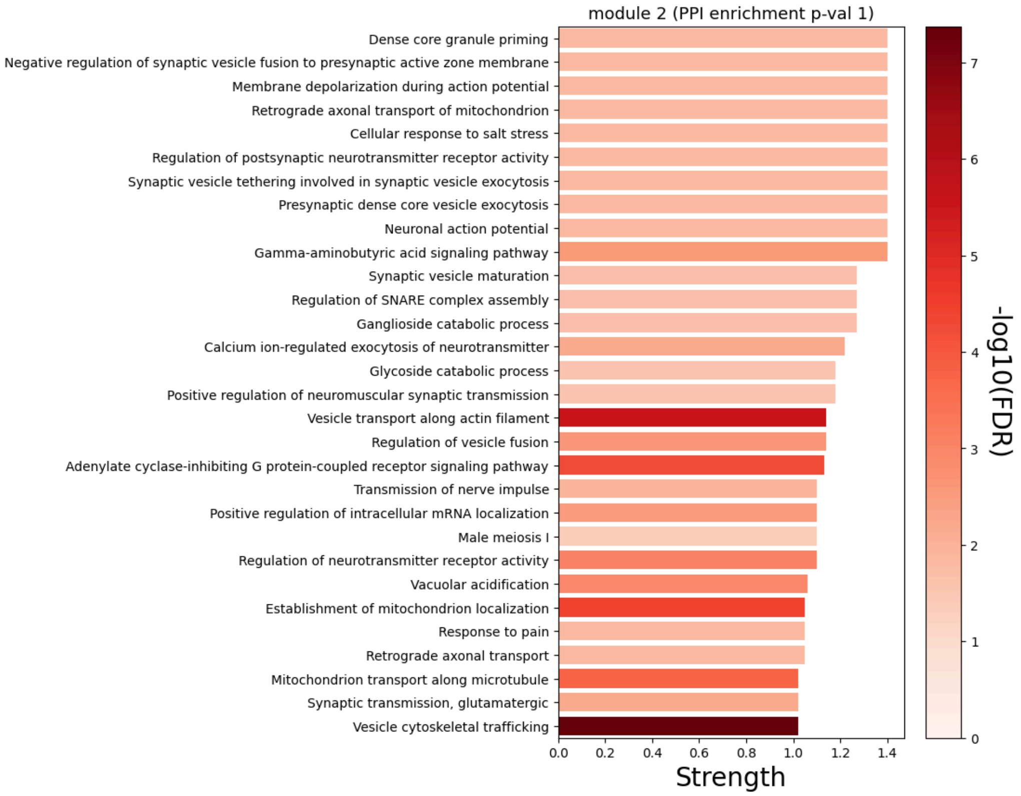

Supplementary Figure 9: Biological process GO enrichment in module 2 (1363 genes).

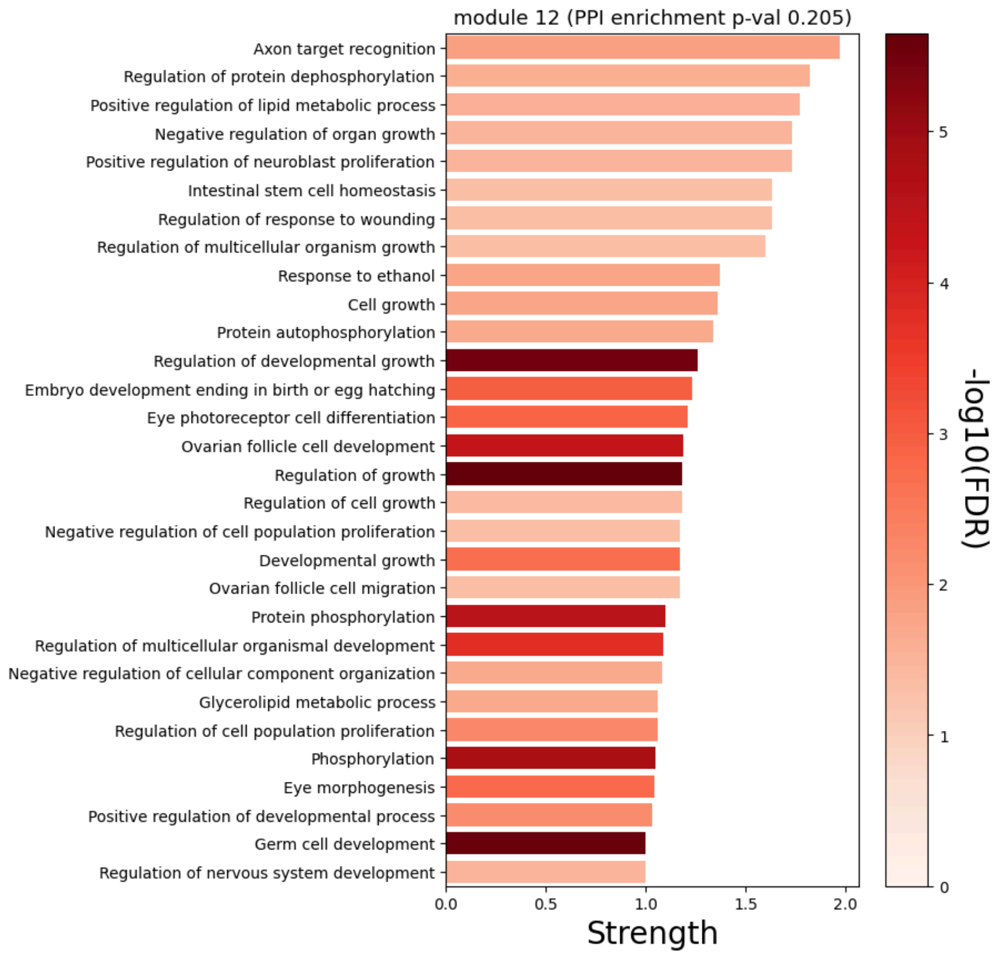

Supplementary Figure 10: Biological process GO enrichment in module 12 (61 genes).

12. Line 297, did the authors mean “<=-0.91” instead of “<=0.91”? Can the author also elaborate somatic cluster -1?

Yes. Thank you.

13. Figure 6 was not mentioned in the text. Did the author mean Figure 6 instead of Figure 5 in line 344 and line 349.

Yes, it is now fixed.

14. Line 425-427, the authors should refer to Figure 6 here.

We added references to figures 5 and 6:

“In our data, the two germline clusters (germ cells A and germ cells B) show lower S0/Autosomal expression (Figure 5 B), which seems to be driven by the enrichment in cells with

S0 downregulation, consistent with a lack dosage compensation compared to somatic clusters. The ATACseq results show a similar pattern, where the germline cells have fewer counts in the S0 region compared to the somatic clusters (Figure 6).”

15. Line 542, the authors should cite the literatures.

Thank you for the remark. We have now added the references to that section.

Have all data underlying the figures and results presented in the manuscript been provided?
Large-scale datasets should be made available via a public repository as described in the *PLOS Genetics* data availability policy, and numerical data that underlies graphs or summary statistics should be provided in spreadsheet form as supporting information.

Reviewer #1: Yes

Reviewer #2: None

Reviewer #3: Yes

PLOS authors have the option to publish the peer review history of their article (what does this mean?). If published, this will include your full peer review and any attached files.

If you choose “no”, your identity will remain anonymous but your review may still be made public.

Do you want your identity to be public for this peer review? For information about this choice, including consent withdrawal, please see our Privacy Policy.

Reviewer #1: No

Reviewer #2: No

Reviewer #3: No

References:

1. Itoh Y, Melamed E, Yang X, Kampf K, Wang S, Yehya N, et al. Dosage compensation is less effective in birds than in mammals. J Biol. 2007 Mar 22;6(1):2.
2. Ellegren H, Hultin-Rosenberg L, Brunström B, Dencker L, Kultima K, Scholz B. Faced with inequality: chicken do not have a general dosage compensation of sex-linked genes. BMC Biol. 2007 Sep 20;5(1):40.
3. Meiklejohn CD, Landeen EL, Cook JM, Kingan SB, Presgraves DC. Sex Chromosome-Specific Regulation in the Drosophila Male Germline But Little Evidence for Chromosomal Dosage Compensation or Meiotic Inactivation. PLOS Biol. 2011 Aug 16;9(8):e1001126.
4. Argyridou E, Parsch J. Regulation of the X Chromosome in the Germline and Soma of Drosophila melanogaster Males. Genes. 2018 May 4;9(5):242.
5. Bett VK, Macon A, Vicoso B, Elkrewi M. Chromosome-Level Assembly of Artemia franciscana Sheds Light on Sex Chromosome Differentiation. Genome Biol Evol [Internet]. 2024 Jan 5 [cited 2024 Feb 5];16(1). Available from: https://dx.doi.org/10.1093/gbe/evae006
6. Anderson J, Henikoff S, Ahmad K. Chromosome-specific maturation of the epigenome in the Drosophila male germline. eLife [Internet]. 2023 Nov 17 [cited 2024 Jan 29];12. Available from: https://elifesciences.org/reviewed-preprints/89373
7. Witt E, Shao Z, Hu C, Krause HM, Zhao L. Single-cell RNA-sequencing reveals pre-meiotic X-chromosome dosage compensation in Drosophila testis. PLOS Genet. 2021 Aug 17;17(8):e1009728.
8. Ota R, Hayashi M, Morita S, Miura H, Kobayashi S. Absence of X-chromosome dosage compensation in the primordial germ cells of Drosophila embryos. Sci Rep. 2021 Mar 1;11:4890.
9. Raz AA, Vida GS, Stern SR, Mahadevaraju S, Fingerhut JM, Viveiros JM, et al. Emergent dynamics of adult stem cell lineages from single nucleus and single cell RNA-Seq of Drosophila testes. Buszczak M, Banerjee U, Buszczak M, Nystul TG, editors. eLife. 2023 Feb 16;12:e82201.
10. Sangrithi MN, Turner JMA. Mammalian X Chromosome Dosage Compensation: Perspectives From the Germ Line. BioEssays. 2018;40(6):1800024.
11. Mattimoe T, Payer B. The compleX balancing act of controlling X-chromosome dosage and how it impacts mammalian germline development. Biochem J. 2023 Apr 26;480(8):521–37.
12. Wei KHC, Chatla K, Bachtrog D. Single-cell RNA-seq of Drosophila miranda testis reveals the evolution and trajectory of germline sex chromosome regulation. PLOS Biol. 2024 Apr 30;22(4):e3002605.
13. Navarro-Costa P, McCarthy A, Prudêncio P, Greer C, Guilgur LG, Becker JD, et al. Early programming of the oocyte epigenome temporally controls late prophase I transcription and chromatin remodelling. Nat Commun. 2016 Aug 10;7(1):12331.
14. Kim YK, Cho B, Cook DP, Trcka D, Wrana JL, Ramalho-Santos M. Absolute scaling of single-cell transcriptomes identifies pervasive hypertranscription in adult stem and progenitor cells. Cell Rep [Internet]. 2023 Jan 31 [cited 2024 Jun 14];42(1). Available from: https://www.cell.com/cell-reports/abstract/S2211-1247(22)01882-4
